# Induction of BDNF Expression in Layer II/III and Layer V Neurons of the Motor Cortex Is Essential for Motor Learning

**DOI:** 10.1523/JNEUROSCI.0288-20.2020

**Published:** 2020-08-12

**Authors:** Thomas Andreska, Stefanie Rauskolb, Nina Schukraft, Patrick Lüningschrör, Manju Sasi, Jeremy Signoret-Genest, Marcus Behringer, Robert Blum, Markus Sauer, Philip Tovote, Michael Sendtner

**Affiliations:** ^1^Institute of Clinical Neurobiology, University Hospital Wuerzburg, 97080 Wuerzburg, Germany; ^2^Department of Biotechnology and Biophysics, Julius-Maximilians-University Wuerzburg, 97074 Wuerzburg, Germany

**Keywords:** BDNF, motor cortex, motor learning, neurotrophic factor, striatum

## Abstract

Motor learning depends on synaptic plasticity between corticostriatal projections and striatal medium spiny neurons. Retrograde tracing from the dorsolateral striatum reveals that both layer II/III and V neurons in the motor cortex express BDNF as a potential regulator of plasticity in corticostriatal projections in male and female mice. The number of these BDNF-expressing cortical neurons and levels of BDNF protein are highest in juvenile mice when adult motor patterns are shaped, while BDNF levels in the adult are low. When mice are trained by physical exercise in the adult, BDNF expression in motor cortex is reinduced, especially in layer II/III projection neurons. Reduced expression of cortical BDNF in 3-month-old mice results in impaired motor learning while space memory is preserved. These findings suggest that activity regulates BDNF expression differentially in layers II/III and V striatal afferents from motor cortex and that cortical BDNF is essential for motor learning.

**SIGNIFICANCE STATEMENT** Motor learning in mice depends on corticostriatal BDNF supply, and regulation of BDNF expression during motor learning is highest in corticostriatal projection neurons in cortical layer II/III.

## Introduction

Adaptive modulation of network activity by synaptic plasticity is a central aspect of learning and memory in the mammalian CNS (Martin et al., 2000). BDNF (Barde et al., 1982) plays a central role in different aspects of neural plasticity and modulation of neuronal network activity (Patterson et al., 1996; Korte et al., 1998; Chen et al., 1999; Minichiello et al., 1999; Schuman, 1999; Messaoudi et al., 2002; Ying et al., 2002; Lu et al., 2014; Edelmann et al., 2015). Plasticity within the corticostriatal network is crucial for learning, adapting, and modulating motor functions and limb movement coordination (West et al., 1990; Fritsch et al., 2010; Chen et al., 2019). In order to regulate this form of plasticity, BDNF is thought to be provided via cortical afferents to striatal neurons (Altar et al., 1997; Conner et al., 1997; Yan et al., 1997; Gorski et al., 2003; Kalivas, 2009; Li et al., 2012; Park et al., 2014). Lack of BDNF supply by conditional postnatal depletion in total brain causes dramatic neurodegenerative changes in the striatum (Rauskolb et al., 2010). The motor cortex is a major source for neocortical input to the striatum. Terminals of these afferents densely innervate the dorsolateral striatum, a region involved in the control of voluntary limb movement (Webster, 1961; Kunzle, 1975; Cospito and Kultas-Ilinsky, 1981; Alexander et al., 1986; Donoghue and Herkenham, 1986; McGeorge and Faull, 1989; West et al., 1990; Kimura et al., 1993; Yin and Knowlton, 2006). Growing evidence proposes an essential function of the motor cortex in learning of motor programs (Grillner, 2015; Kawai et al., 2015). This also implicates a role of cortical BDNF in modulating corticostriatal network activity (Sheng et al., 2019) and motor skill acquisition (Fritsch et al., 2010; Chen et al., 2019). Efficiency in acquiring motor skills is higher in young compared with older individuals in a variety of motor tasks (Voelcker-Rehage and Alberts, 2007; Fraser et al., 2009; King et al., 2013). Interestingly, this correlates with decreasing BDNF levels during aging (Lommatzsch et al., 2005; Erickson et al., 2010). Cortical BDNF expression was initially identified in layers II/III, V, and VI (Altar et al., 1997; Conner et al., 1997; Yan et al., 1997; Gorski et al., 2003; Kalivas, 2009; Li et al., 2012; Park et al., 2014) and appears directly linked to neuronal activity (i.e., through physical activity) (Neeper et al., 1995, 1996; Griesbach et al., 2004; Rasmussen et al., 2009; Chen et al., 2019).

Not much is known about the identity of BDNF-expressing corticostriatal neurons and the relation of motor activity, motor skill learning, and BDNF expression in different layers of the motor cortex. We investigated which types of corticostriatal projection neurons express BDNF, how this expression is altered during postnatal development, and whether physical activity modulates BDNF expression in these neurons. Using refined immunohistochemistry methods, we found that postnatal BDNF protein levels in anterior cortex and motor cortex peak around P21 and then decrease during adulthood. Cortical BDNF expression increases after motor exercising in a running wheel, especially in layers II/III of the motor cortex. This effect was not observed in corticothalamic neurons. Cortical BDNF depletion results in deficits in motor skill acquisition. These findings point to a role of BDNF expression in layers II/III and V of the motor cortex in modulation of corticostriatal networks for motor learning.

## Materials and Methods

### 

#### Mouse lines

Brain tissue was dissected from P21, P56, and P84 male mice for IHC and ELISA analyses from WT (C57Bl6/J, RRID:IMSR_JAX:000664), *Bdnf-Myc* (Matsumoto et al., 2008), and either *NFL-Cre^wt/tg^/BDNF^fl/wt^* or *NFL-Cre^wt/tg^/BDNF^fl/KO^* mice. Conditional BDNF KO mice were generated by crossing mice expressing cyclic recombinase (CRE) under control of the neurofilament light chain promoter (NF-L) (Schweizer et al., 2002) with mice carrying a *bdnf* exon V, flanked by two loxP sites, on one allele and a neomycin cassette in the 5′ coding region of exon V on the second allele (Rauskolb et al., 2010). All experiments were approved by a license for animal testing (RUF-55.2.2-2532-2-728-21) and performed in accordance with the supervision through local veterinary authority (Veterinaeramt der Stadt Wuerzburg) and Committee on the Ethics of Animal Experiments (i.e., Regierung von Unterfranken, Wuerzburg).

#### Stereotaxic surgery and neuronal tracing

Male C57Bl-6/J mice (P21 and P84) were anesthetized with isoflurane (2% induction, 1.2%-1.5% maintenance in 95% oxygen) and placed in a stereotactic apparatus (Kopf 992, Neurostar). Craniotomies were performed using an electric drill (200-400 µm) at the position of the desired target region (dorsolateral striatum AP: 0.6 mm; ML: 1.7 mm; DV: 3 mm from bregma). Calibrated glass pipettes (5 µl microcapillary tube; Sigma Millipore), which were cut with a pulled-glass capillary (PC-100; Narishige) and connected to a pressure ejection system (PDES-02XD; NPI), were inserted into the target region at a speed of 0.8 mm/min. Flow rate of injection was kept at 0.33 nl/min. Fluorescent latex tracer beads were injected at a total volume of <1 µl into the dorsolateral striatum of the right hemisphere. The pipette was then removed stepwise at 0.8 mm/min. The wound was closed and treated with Cutasept (self-made). After surgery, mice were given meloxicam (12 mg/kg, s.c.) and were allowed to recover for at least 24 h before offering a running wheel for voluntary exercise for 72 h before transcardial perfusion for IHC. Mice were weighed daily to monitor their recovery.

#### Voluntary physical exercise

Male C57Bl6/J mice (P21 and P84) were allowed voluntary access to a running wheel for 72 h, connected to a digital counting device. The rotations were documented for each individual animal and used for calculation of the average distance run. C57Bl6/J mice that obtained the tracer injection but no access to a running wheel were used as sedentary controls.

#### Preparation of tissue for immunostaining

Mice were deeply anesthetized with 120 mg/kg ketamine hydrochloride and 16 mg/kg xylazine hydrochloride in 0.4-0.6 ml 1× PBS and transcardially perfused through the left ventricle. Blood vessels were flushed with 1× PBS, 0.4% heparin for 2-3 min. Fixative perfusion was performed with 2%-4% PFA, pH 6.0, in PB for ∼8 min. Subsequently, brains were removed from the skull and allowed for postfixation in 2%-4% PFA at 4°C for 0.5-2 h. Brains were then washed in 1× PBS and embedded in 6% agarose; 20-40 µm free-floating, coronal brain sections were obtained using a Vibratome VT1000S (RRID:SCR_016495; Leica Microsystems) and stored in Cryoprotection Anti-Freeze Buffer (1× PBS, glycerol, ethylene glycol) at −20°C.

#### Antibodies for immunostaining

BDNF was detected using different mouse monoclonal anti-BDNF antibodies directed against the mature form of BDNF: mAb#9 (RRID:AB_2617199) (Kolbeck et al., 1999), mAb#3B2 (Icosagen #329-100), mAb#3C11 (Icosagen #327-100) and mAb#4C8 (Icosagen #328-100). BDNF-Myc was visualized with rabbit polyclonal (Abcam, Ab9106, RRID:AB_307014; Santa Cruz Biotechnology, SC789, RRID:AB_631274) or goat polyclonal (Abcam, 9132, RRID:AB_307033) anti c-Myc antibodies. ProBDNF was visualized with a rabbit polyclonal antiserum against the prodomain of human pro-BDNF (Alomone Labs, #ANT-006, RRID:AB_2039758) (Dieni et al., 2012). Presynaptic corticostriatal terminals were labeled with rabbit polyclonal antibodies against vesicular glutamate transporter 1 (VGluT1) (Synaptic Systems, #135302, RRID:AB_887877). Nigrostriatal projections were identified with a chicken polyclonal antibody against TH (Millipore, #AB9702, RRID:AB_570923). Cortical neurons were stained with the following antibodies: layer II/III, rabbit anti-CDP (Cux-1; Santa Cruz Biotechnology, SC13024, RRID:AB_2261231) and layer V/VI, rat anti-CTIP-2 (Abcam, Ab18465, RRID:AB_2064130).

The following secondary antibodies were used: donkey anti-mouse DyLight549 (Jackson ImmunoResearch Laboratories; 715-505-150), donkey anti-mouse DyLight550 (Thermo Fisher Scientific; #SA5-10167, RRID:AB_2556747), donkey anti-rabbit Alexa488 (Jackson ImmunoResearch Laboratories; 711-545-152, RRID:AB_2313584), Cy3 (Jackson ImmunoResearch Laboratories; 711-165-152, RRID:AB_2307443), Alexa647 (Jackson ImmunoResearch Laboratories; 711-605-152, RRID:AB_2492288), DyLight649 (Jackson ImmunoResearch Laboratories; 711-495-152, RRID:AB_2315775), donkey anti-goat Cy2 (Jackson ImmunoResearch Laboratories; 705-225-003, RRID:AB_2340420), Alexa488 (Jackson ImmunoResearch Laboratories; 705-545-147, RRID:AB_2336933), Alexa647 (Jackson ImmunoResearch Laboratories; 705-605-003, RRID:AB_2340436), donkey anti-chicken Alexa488 (Jackson ImmunoResearch Laboratories; 703-545-155, RRID:AB_2340375), Alexa647 (Jackson ImmunoResearch Laboratories; 703-605-155, RRID:AB_2340379), and donkey anti rat Dylight405 (Jackson ImmunoResearch Laboratories; 712-475-153, RRID:AB_2340681).

#### Immunohistochemistry

Free-floating vibratome sections were washed in 1× PBS. Blocking and permeabilization were performed as one step using 1× PBS, 0.3% Triton X-100, 0.1% Tween 20, and 10% normal donkey serum for 2 h. Primary antibodies were diluted in permeabilization and blocking buffer and incubated at a final concentration between 0.5 and 1.0 µg/ml in the presence of 0.01% NaN_3_ on a shaker at 4°C for 72 h. Afterwards, the slices were washed in 1× PBS, 0.1% Triton X-100, 0.3% Tween 20. Secondary antibodies were diluted in permeabilization and blocking buffer at a final concentration of 0.625 µg/ml. After secondary antibody incubation, the brain slices were extensively washed with 1× PBS, 0.1% Triton X-100, 0.3% Tween 20. Nuclei were stained with 0.4 µg/ml DAPI. After DAPI incubation, sections were washed twice in 1× PBS before they were rinsed in dH_2_O and finally mounted on SuperfrostPlus glass slides (25 × 75 × 1.0 mm, Thermo Fisher Scientific; #J1800AMNZ) using MERCK-FluorSave reagent (Merck; #345789-20ML).

#### Confocal microscopy

Coronal brain slices were analyzed with an Olympus FluoView 1000 confocal laser microscope equipped with the following objectives: 10× (NA: 0.25), 20× (NA: 0.75), 40× (oil differential interference contrast, NA: 1.30), or 60× (oil differential interference contrast, NA: 1.35). Images were obtained with the corresponding Olympus FV10-ASW (RRID:SCR_014215) imaging software for visualization and image acquisition in a single-channel scan mode as *z* stacks, using 405, 473, 559, and 633 nm lasers. The resulting images (Olympus .oib format) were processed using ImageJ (RRID:SCR_003070) and projected as either maximum or average intensity (indicated in the figure legends for all images shown in this study). Superresolution images were obtained with an Elyra S.1 structural illumination microscopic (SIM) setup (Carl Zeiss) and ZEN 2.1 SP-1 image acquisition software (Carl Zeiss). Brightness and contrast were adapted, as indicated in Table 1. The γ correction was not changed in any case. Finally, the data were transferred into tif format, arranged with Adobe Illustrator software (RRID:SCR_010279), and saved as 300 dpi png and tif files.

**Table 1.** Image preparation

Figure	Projection mode	Type	405 nm	488 nm	550 nm	647 nm
1*A*	2D merge	0-255	DAPI	CTIP-2 (10-160)	BDNF	
		(8 bit)	(no change)		(34-138)	
1*B*	Maximum intensity projection	0-4095	DAPI		BDNF	
		(12 bit)	(2000-4095)	(200-2503)		
1*C* WT CTR	Average intensity projection	0-4095	DAPI mAb#9	BDNF		
		(12 bit)	(400-4095)	(700-3003)		
			DAPI mAb#4C8			
			(200-2002)			
			DAPI mAb#3C11, 3B2			
			(300-2503)			
1*C* BDNF KO	Average intensity projection	0-4095	DAPI (300-2503)		BDNF	
		(12 bit)		(700-3003)		
1*D*	Maximum intensity projection	0-4095	DAPI		BDNF	Myc (AB9106)
		(12 bit)	(250-2503)	(200-750)	(200-450)	
					Myc (SC789)	
					(150-500)	
2*B*	Maximum intensity projection	0-4095	DAPI		BDNF	
		(12 bit)	(300-4095)	(1401-3804)		
3*A*	2D merge	0-255		CTIP-2	BDNF	
		(8 bit)	(35-140)	(65-170)		
3*D*	Maximum intensity projection	0-4095	DAPI	Tracer		Cux-1
		(12 bit)	(1802-4095)	(2002-4095)	(600-2002)	
3*E*	Maximum intensity projection	0-4095	CTIP-2	Tracer	BDNF	Cux-1
		(12 bit)	(400-2002)	(901-4095)	(1601-4095)	(901-4095)
4*A*	Maximum intensity projection	0-4095		Tracer	BDNF	Cux-1
P21 CTR		(12 bit)	(1201-4095)	(1301-4095)	(600-3003)	
4*A*	Maximum intensity projection	0-4095		Tracer	BDNF	Cux-1
P21 RW		(12 bit)	(600-3003)	(1301-4095)	(600-4095)	
4*A*	Maximum intensity projection	0-4095		Tracer	BDNF	Cux-1
P84 CTR		(12 bit)	(1201-4095)	(1301-4095)	(600-4095)	
4*A*	Maximum intensity projection	0-4095		Tracer	BDNF	Cux-1
P84 RW		(12 bit)	(600-3003)	(1301-4095)	(600-4095)	
5*A*	Maximum intensity projection	0-4095	CTIP-2	Tracer	BDNF	
P21 CTR		(12 bit)	(1201-3504)	(600-3003)	(1301-4095)	
5*A*	Maximum intensity projection	0-4095	CTIP-2	Tracer	BDNF	
P21 RW		(12 bit)	(399-1700)	(600-3003)	(600-2302)	
5*A*	Maximum intensity projection	0-4095	CTIP-2	Tracer	BDNF	
P84 CTR		(12 bit)	(350-1301)	(600-3003)	(800-2302)	
5*A*	Maximum intensity projection	0-4095	CTIP-2	Tracer	BDNF	
P84 RW		(12 bit)	(350-1301)	(600-3003)	(600-1802)	
6*A*	Maximum intensity projection	0-4095		CTIP-2	BDNF	
P21 CTR		(12 bit)	(700-4095)	(1201-4095)		
6*A*	Maximum intensity projection	0-4095		CTIP-2	BDNF	
P21 RW		(12 bit)	(700-4095)	(1201-4095)		
6*A*	Maximum intensity projection	0-4095		CTIP-2	BDNF	
P84 CTR		(12 bit)	(700-4095)	(1201-4095)		
6*A*	Maximum intensity projection	0-4095		CTIP-2	BDNF	
P84 RW		(12 bit)	(700-4095)	(1201-4095)		
7*A* confocal	Maximum intensity projection	0-4095	DAPI	TH	BDNF	VGluT1
		(12 bit)	(100-2002)	(150-2002)	(400-1697)	(500-4095)
7*A*	Maximum intensity projection	0-65535		TH	BDNF	VGluT1
SIM		(16 bit)	(1921-25624)	(3843-26457)	(2562-33888)	
7*B*	Maximum intensity projection	0-65535		TH	BDNF	VGluT1
SIM		(16 bit)	(1921-25624)	(3843-26457)	(2562-33888)	
7*E*	Maximum intensity projection	0-65535			BDNF	VGluT1
P21 sedentary SIM		(16 bit)	(1300-7000)	(1300-25.000)		
7*E*	Maximum intensity projection	0-65535			BDNF	VGluT1
P21 running-wheel SIM		(16 bit)	(600-12000)	(2500-33.000)		
3-1*A*, left	Maximum intensity projection	0-4095	DAPI		BDNF	pro-BDNF
		(12 bit)	(600-4095)	(900-4095)	(500-2500)	
3-1*A*, right	Maximum intensity projection	0-4095	DAPI		BDNF	pro-BDNF
		(12 bit)	(300-4095)	(500-4095)	(600-3500)	
3-1*B*, left	Maximum intensity projection	0-4095	DAPI		BDNF	pro-BDNF
		(12 bit)	(600-4095)	(900-4095)	(500-2500)	
3-1*B*, right	Maximum intensity projection	0-4095	DAPI		BDNF	pro-BDNF
		(12 bit)	(300-4095)	(500-4095)	(600-3500)	
3-1*C*	Maximum intensity projection	0-4095			BDNF	pro-BDNF
		(12 bit)	(900-4095)	(400-3500)		
3-1*D*, P21 sedentary	Maximum intensity projection	0-4095	CTIP-2		BDNF	pro-BDNF
		(12 bit)	(800-2500)	(900-4095)	(400-3500)	
3-1*D* P28	Maximum intensity projection	0-4095	CTIP-2		BDNF	pro-BDNF
*NFL-Cre BDNF^fl/ko^*		(12 bit)	(250-1000)	(900-4095)	(400-2500)	
4-3*A*	Maximum intensity projection	0-4095			BDNF	Cux-1
		(12 bit)	(1000-4095)	(350-4095)		
4-3*B*	Maximum intensity projection	0-4095	CTIP-2		BDNF	
		(12 bit)	(425-2500)	(1000-4095)		

*^a^*Overview of the number of independent experiments, animal counts (including gender and age), number of images and cell counts.

**Table 2.** Transparent reporting*^b^*

Experiment	Total no. of experiments	Total no. of individualanimals			Total no. ofconsidered images	
BDNF detection in hippocampusand striatum Figure 1*A–D*						
Hippocampus (WT) Figures 1*A–C*, 2*B*	45	105			272	
Hippocampus (*BDNF-myc*) Figure 1*D*	11	2			21	
Hippocampus (BDNF-ko) Figure 1*B,C*	9	6			20	
Hippocampus monoclonalantibodies Figure 1*C*	4	8			38	
Striatum Figure 7*A,B*	6	6			32	
Striatum statistical analysisFigure 7*C*	1	1			15	
Experiment	Total no. ofexperiments	Total no. of individualanimals			Total no. ofconsidered images	Total no. of countedBDNF-positive cells
BDNF vs pro-BDNF-IR inhippocampus and cortex						
Figure 3-1						
3 week CTR hippocampus	5	2			20	none
4 week BDNF-ko hippocampus	4	1			10	none
3 week CTR cortex	3	2			17	none
4 week BDNF-ko cortex	2	1			12	none
Experiment	Total no. ofexperiments	Total no. of individualanimals		Total no. of tracedanimals	Total no. ofconsidered images	Total no. of countedBDNF-positive cells
BDNF detection in cortical neurons						
Figures 3–6						
Figures 4-2, 4-3						
3 week CTR layer II/III	5	10		4	65	1353
3 week RW layer II/III	5	8		5	51	1651
12 week CTR layerII/III	4	8		5	47	503
12 week RW layerII/III	3	6		6	40	1114
3 week CTR layer V	5	10		4	64	1400
3 week RW layer V	5	8		5	46	1541
12 week CTR layer V	4	8		5	42	438
12 week RW layer V	3	6		6	40	813
3 week CTR layer VI	4	10		0	52	2194
3 week RW layer VI	4	9		0	48	1930
12 week CTR layer VI	3	9		0	45	1572
12 week RW layer VI	2	8		0	50	1814
Experiment	Total no. ofexperiments	Total no. of individualanimals			Total no. ofconsidered images	Total no. of analyzedBDNF-positive cells
BDNF-IR intensity						
Figures 4, 5*D*, 6*C*						
3 week CTR layer II/III	7	10			90	559
3 week RW layer II/III	5	8			50	384
12 week CTR layerII/III	4	6			72	369
12 week RW layerII/III	3	6			42	288
3 week CTR layer V	7	10			100	680
3 week RW layer V	4	7			48	396
12 week CTR layer V	3	6			56	334
12 week RW layer V	2	6			36	215
3 week CTR layer VI	4	10			60	585
3 week RW layer VI	3	7			42	421
12 week CTR layer VI	2	7			42	386
12 week RW layer VI	1	4			24	225
Experiment	Total no. ofexperiments	Total no. of individualanimals	Total no. of consideredimages	Total no. of countedBDNF-positive cells		
BDNF detection incortical neurons						
Figure 4-3*A*,*B*						
3 week CTR layer II/III	1	1	3	None		
3 week RW layer II/III	1	1	4	None		
4 week cBDNF ko layer II/III	1	1	2	None		
3 week CTR layer V	1	1	3	None		
3 week RW layer V	1	1	2	None		
4 week cBDNF ko layer V	1	1	3	None		
Experiment	Total no. ofexperiments	Total no. of individualanimals	Total no. of animalsconsidered for figure			
BDNF qRT-PCR Figure 4-3*C*						
3 week CTR layer II/III	2	3	2	None		
3 week cBDNF ko layer II/III	2	1	1	None		
3 week CTR layer V	2	3	2	None		
3 week cBDNF ko layer V	2	1	1	None		
Experiment	Hippocampus	Striatum		Anterior cortex		Cerebellum
BDNF-ELISA Figure 1*E*						
WT no. of individual animals	3	5		5		5
*NFL-Cre BDNF^fl/ko^* no. of individualanimals	2	2		2		2
Experiment	Hippocampus	Striatum		Cortex anterior		Cortex posterior
BDNF-ELISA Figure 2*A*						
P10 no. of individual animals	3	2		3		3
P14 no. of individual animals	3	3		3		3
P20 no. of individual animals	3	3		3		3
P84 no. of individual animals	4	4		4		4
BDNF Western blot detectionFigure 7*D*	Total no. ofexperiments	Total no. of individualanimals		Total no. of consideredanimals in Figure		
Anterior cortex vs striatum:sedentary, runners, cBDNFKO Figure 7*D*	11	13 sedentary		9 sedentary		
		13 runner		9 runners		
		2 *NFL-Cre BDNF^fl/ko^*		2 *NFL-Cre BDNF^fl/ko^*		
Open Field	Total no. of mice					
Figure 8*A*,*B*						
WT	4 females, 1 male					
*NFL-Cre BDNF^fl/wt^*	4 females, 1 male					
Y-Maze	Total no. of mice					
Figure 8*C–E*						
WT	8 females, 2 males					
*NFL-Cre BDNF^fl/wt^*	4 females, 1 male					
Rotarod 8-week-old mice	Total no. of mice	Rotarod 34-week-oldmice	Total no. of mice			
Figure 8*F*,*G*		Figure 8*H*,*I*				
WT	7 males	WT	4 females, 1 male			
*NFL-Cre BDNF^fl/wt^*	6 males	*NFL-Cre BDNF^fl/wt^*	4 females, 1 male			
Irregular ladder rung Figure 9	Total no. of mice	Total no. of mice tested day 1		Total no. of mice tested day 2		Total no. of runsper day
WT	7	7		6		5
*NFL-Cre BDNF^fl/wt^*	6	6		6		5

*^b^*Image preparation was performed using ImageJ software. Changes were made exclusively on brightness and contrast. γ values were not altered in any case.

#### BDNF Western blot analysis

Mice were killed with CO_2,_ and body weight was measured before animals were decapitated. The brains were prepared and transferred to either ice-cold 1× PBS or 1× HBSS. Anterior cortex, posterior cortex, hippocampus, striatum, and cerebellum of P21 C57Bl6/J sedentary and wheel-runner (72 h) or *NFL-Cre BDNF^fl/ko^* mice were dissected and immediately frozen in liquid N_2_. Tissue was lysed in 0.05 m sodium acetate, 1 m NaCl, 0.1% Triton X-100, pH 4.0, containing protease inhibitor (Roche Diagnostics, #11697498001 or #11836153001). A Hielscher sonifyer was used for tissue lysis. Samples were then subjected to 80 min centrifugation at 20,000 × *g* (4°C), and supernatants were collected. Protein content was measured using a Pierce Protein Research BCA kit (Thermo Fisher Scientific, #23225). Either 30 or 40 µg of protein was diluted in 4× Laemmli buffer and boiled at 99°C for 5 min before being applied to 18% PAA gels. Gel electrophoresis was performed at constant U = 120 V for 120-140 min before blotting on PVDF membrane at U = 120 V, I = 0.40 A, P = 50 W for 40 min. PVDF membranes were blocked with 1× TBST, 7% BSA (fraction V, Applichem, #A1391,0100). BDNF was detected using mouse mAb 3C11 (Icosagen, #327-100) and horse anti-mouse HRP-linked secondary antibody (Cell Signaling Technology, #7076, RRID:AB_330924). BDNF was detected using ECL Western blotting Detection kit (Millipore, Immobilon Western HRP Substrate Luminol Reagent #WBKLS0500). After detection of BDNF, HRP was inactivated by washing 15 min in 1× TBST, 0,1% NaN_3_, followed by extensive rinsing in 1× TBST. Mouse mAb anti-cytochrome C (HRP-linked; Santa Cruz Biotechnology, A-8 #sc-13156, RRID:AB_627385) was diluted in 1× TBST and incubated at 4°C overnight. CytC was detected with Pierce ECL Western blotting substrate (Thermo Fisher Scientific, #32106).

#### Preparation of tissue for BDNF ELISA

Mice were killed with CO_2_, and body weight was measured before animals were decapitated. The brains were prepared and transferred to either ice-cold 1× PBS or 1× HBSS and cortex, hippocampus, striatum, and cerebellum, as well as the brainstem with midbrain were dissected and collected in 2.0 ml Eppendorf tubes. The samples were then immediately frozen in liquid nitrogen and stored at −80°C. Tissue extraction was performed according to the BDNF Western blot procedure. Supernatants containing the protein fraction were collected in 2.0 ml Eppendorf tubes. BDNF immunoassay was performed according to the original protocol published previously (Kolbeck et al., 1999; Rauskolb et al., 2010). Samples for the BDNF standard curve contained recombinant BDNF at concentrations of 0.0, 0.2, 0.4, 0.8, 1.6, 3.2, 6.4, and 12.8 ng/ml. All samples and samples for standard curve were applied as duplicates of 50 µl volume to the pretreated ELISA plate. Peroxidase-coupled anti-BDNF antibodies were detected using peroxidase substrate (ELISA substrate, Roche Diagnostics). Chemoluminescence was detected with an ELISA reader (Inifinite M200 Pro, TECAN). The background luminescence (0.0 ng BDNF) was subtracted as background noise from luminescence values of all tissue extracts. Relative BDNF levels in tissue extracts from P10, P14, P20, P84, or P21 WT versus *NFL-Cre^Wt/Tg^/BDNF^fl/KO^* mice were determined as percent of the mean hippocampal luminescence value in P20 or P21 mice, according to the corresponding experimental setup. Microsoft Excel and GraphPad Prism 6.0 software (RRID:SCR_002798) was used for calculation and visualization.

#### Laser micro-dissection (LMD)

Freshly dissected anterior cortex prepared from P21 C57/Bl6 sedentary and wheel runner (72 h) or *NFL-Cre BDNF^fl/ko^* mice was embedded in Tissue-Tek O.C.T. Compound (Science Services, SA62550-01) and frozen in liquid nitrogen; 20 µm coronal brain sections were prepared with a Leica Kryostat (Leica Microsystems, CM1950, RRID:SCR_018061), mounted on polyethylene naphthalate object slides, fixed in RNase free 75% EtOH for 3 min, and dried at 40°C for 10 min. These sections were quickly stained with 0.02% Toluidine blue to visualize brain structures. Cortical layers II/III and V were dissected at 5× magnification using a Leica Microsystems DM6000B LMD setup equipped with a Leica CTR6500 laser. Tissue was collected in 0.2 ml RNase free safe lock PCR tubes and processed immediately for RNA purification.

#### RNA purification and qRT-PCR

RNA purification was performed using the Arcturus PicoPure RNA Isolation Kit (#KIT0202, KIT0204), including digestion of genomic DNA using a RNase free DNase set (QIAGEN, #79254). RNA concentration was measured with a Nanodrop Spectrophotometer (Peqlab; ND-1000, RRID:SCR_016517); 20 ng purified RNA was used for reverse transcriptase-mediated cDNA synthesis, using the Thermo Fisher Scientific First Strand cDNA Synthesis Kit (#K1612). cDNA was diluted 1:5 in RNase free H_2_O, and 2 µl cDNA was used for qRT-PCR using a Roche Diagnostics LightCycler 96 with the following primers against total BDNF (forward: 5'-AAATTACCTGGATGCCGCAAAC-3'; reverse: 5'-CGCTGTGACCCACTCGCTAA-3') and mouse GAPDH (forward: 5'-GCAAATTCAACGGCACA-3'; reverse: 5'-CACCAGTAGACTCCACGAC-3'). Results were exported to Microsoft Excel and analyzed with GraphPad Prism 6.0 for statistical analysis. BDNF values were normalized to GAPDH, and BDNF mRNA levels were calculated in percent of mean BDNF mRNA levels in P21 sedentary mice.

#### Quantification of BDNF expression in cortex and striatum

##### BDNF expression in cortical neurons

Analysis was performed nonblinded and manually by the experimenter. BDNF-positive cells were counted in each image of a stack of 10-13 images and investigated for overlap with layer-specific markers (Cux-1, CTIP-2) or fluorescent tracer beads. Forty to 65 images from at least 5 animals per condition were examined in at least three independent IHC approaches. As an unbiased control of the manually scored results, all images were automatically analyzed using the 3D object counter tool in ImageJ with a defined threshold. The threshold was set 25% higher than the mean intensity of the maximum intensity projection for each image. However, this tool produced false-positive results in P84 animals (see Extended Data Fig. 4-4 and Fig. 4-8) because of lipofuscin granules or when blood vessels exhibited autofluorescence. In order to validate the experimenter's manual analysis and to assess interrater reliability, 4 independent blinded experts counted BDNF-positive cells. A first round with three randomly chosen samples served as test for counting. Three different randomly chosen samples per condition were counted in a second round and used for the analysis. Interrater reliability was calculated with correlation analysis, using GraphPad Prism software. Pearson's correlation coefficient *R* was 0.9475 for layer II/III datasets, 0.9479 for layer V data, and 0.7468 for layer VI data. For BDNF-IR measurement, two images of a stack that did not show the same cells were chosen accordingly to all compared images and a total of maximum 10 BDNF-positive cells per image were measured, using a quadratic ROI of defined size. To obtain the BDNF-IR per cell the mean IR of 5 BDNF-negative cells (using the same ROI) was subtracted from each BDNF-positive cell. Negative values for BDNF-IR intensity were excluded from the quantification.

##### BDNF presence in VGluT1 or TH presynaptic terminals in the striatum

For quantification, a total of four confocal and 11 SIM images, obtained from the same animal, were analyzed. The total number of VGluT1 or TH presynaptic structures was counted manually, and the percentage of VGluT1/BDNF or TH/BDNF double-positive structures was calculated for each individual image. As a control, ImageJ was used to calculate the Costes *p* value (Costes et al., 2004) as an unbiased measure for true colocalization. Alternatively, four independent confocal images were analyzed automatically for Costes *p* value and Pearson's *R* value (no threshold) as a measure for the amount of colocalization between BDNF and either VGluT1 or TH.

#### Statistical analysis

The number of experiments was designed at the planning stage, based on numbers of independent experiments that are commonly used for these types of experiments in this research field. All datasets from the quantification of cortical BDNF expression were analyzed for normality by Shapiro–Wilk test. Afterward, *t* test was performed between pairs (normal distribution) or for more than two groups one-way/two-way ANOVA and Tukey multiple comparison test (normal distribution), or Kruskal–Wallis, Mann–Whitney, or Friedman test (no normal distribution) was performed, using GraphPad Prism software. Correlation analysis for interrater reliability was performed with correlation or linear regression analysis using GraphPad Prism software. The type of statistical test is indicated in each figure.

#### Behavioral analysis

##### Open Field test

Individual mice were placed in the middle of a white polyvinyl chloride box (48 × 48 × 50 cm) evenly illuminated with 40-45 lux. The floor of the box was divided into different fields of interest to monitor the abode of individual mice. A webcam (Logitech) was positioned above the box to monitor the track of mice for 10 min. Tracks were recorded, analyzed, and exported as Excel files, using Video Mot Software (TSE). The following parameters were measured and compared between the center of the arena (24 × 24 cm) and the periphery: total distance traveled over time, time spent in the center, and distance traveled in the center. Final statistical analysis was performed using GraphPad Prism software.

##### Rotarod test

A programmable, digital rotarod machine (Ugo Basile) with timers and falling sensors was used to test individual mice. Before the training sessions, mice were allowed to habituate to stay on the rod for 1 min every day. The initial speed, used for habituation, was set to 10 rpm. Once started, the speed was increased to 40 rpm within 15 s, followed by a decrease back to 10 rpm, again within 15 s. Afterward, the rod stopped and inverted its rotation direction, followed by another sequence of acceleration, deceleration, and inversion of the rotation direction. The test was stopped either when individual mice fell off the rod or after a maximum of 100 s. Accelerated, rocking rotarod was performed on 4 subsequent days. For all tests, the latency to fall off the rod was measured in seconds. Two sets of BDNF WT and *NFL-Cre BDNF^fl/wt^* mice were tested. The first set was exclusively male mice at an age of ∼8 weeks; the second set consisted of 4 females and 1 male mouse each, for both groups at an age of ∼34 weeks.

##### Y-Maze test

The Y-Maze test consisted of a Y-shaped polyvinyl chloride box of 14 cm height and with three individual arms of 35 cm length that can be separated from each other by insertion of removable doors. A webcam (Logitech) was positioned above the box to monitor the track of mice. For calculations, the Y-Maze was subdivided into four regions: (1) start-arm (A), (2) selectable arm (B), (3) selectable arm (C), and (4) center. Spontaneous alternation and spatial reference memory were monitored using an adapted protocol of Kraeuter et al. (2019). Mice at an age of 8-10 months were allowed to enter the Y-Maze with all arms open during an initial “reference run” for 8 min. Spontaneous alternations and arm entries were documented, and the percentage of spontaneous alternations was calculated using the following formula:

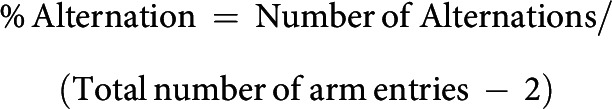
 For spatial reference memory training, mice completed a total of four training runs of 5 min duration with a 1 h intertrial interval on 2 consecutive days. During the training sessions, one arm (B or C) was closed. The final training session was followed by a 1 h intertrial interval and a 5 min, monitored test-run, with all arms open. The time spent in, distance traveled in, and entries into arm B and C were calculated as percent of total time, distance, or arm entries, for each minute of the 5 min test-run. Afterward, the values for time, distance, and arm entries of the initial “reference run” were subtracted from the test run values to calculate a preferred selection of one arm after the training sessions.

##### Ladder rung walking task

The ladder rung walking test was adapted from previous studies (Metz and Whishaw, 2002, 2009; Farr et al., 2006). The apparatus consisted of 0.7 m sidewalls made of acrylic glass. Metal rungs (1-2 mm diameter) were inserted to create a grid with a minimum distance of 0.5 cm between rungs. The ladder was placed 20 cm above the ground with the home cage at the end. Three-month-old mice were placed on the opposite side to enter the rung. To prevent the animal from turning around, the width of the alley was 2.8 cm. For the irregular pattern, the distance of the rungs varied systematically from 0.5 to 4 cm. Two templates of irregular rung patterns were used on 2 subsequent days using one pattern per day. For video recording, a camera was positioned in an ∼30° angle below the rung enabling to monitor all four limbs. Data were analyzed manually, using a 7 category foot fault scoring according to Metz and Whishaw (2002).

## Results

### Characterization of monoclonal antibodies for quantification and immunodetection of BDNF in postnatal mouse brain

**Figure 1.**
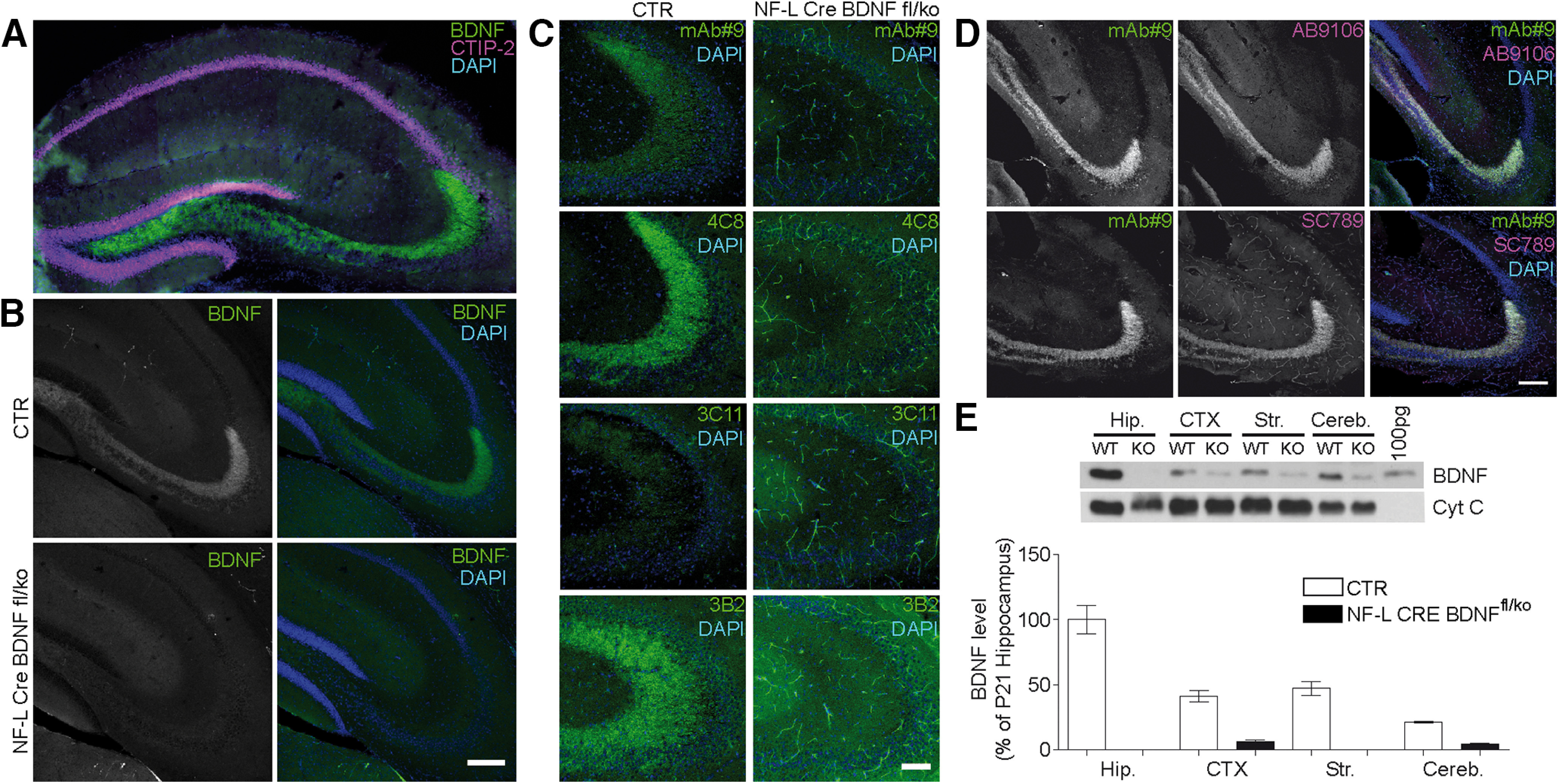
Detection and quantification of BDNF in mouse hippocampus with different monoclonal antibodies. ***A***, BDNF-IR using mAb#9 antibody in P21 WT C57Bl6/J hippocampus. CTIP-2 expression highlights granule cells in the dentate gyrus and pyramidal projection neurons in cornu ammonis (CA) 1-3. ***B***, BDNF-IR in hippocampal mossy fiber projections of 8 week male WT C57Bl6/J versus *NFL-Cre BDNF^fl/ko^* hippocampus, using mAb#9. ***C***, Specificity of 4 independent BDNF antibodies in CA3 mossy fiber terminals of WT C57Bl6/J (left column) and *NFL-Cre BDNF^fl/ko^* (right column). Clone 3C11 failed to detect endogenous BDNF. 3B2 produced high background in BDNF depleted hippocampal sections. The signal-to-noise ratio appeared best with mAb#9 and also with 4C8, which showed intense BDNF-IR. ***D***, Double staining of BDNF and Myc in hippocampus of 8 week male *BDNF-myc* mice. Myc was costained with two independent polyclonal myc antibodies (Abcam, AB9106; Santa Cruz Biotechnology, SC789). ***E***, BDNF protein levels in different CNS mouse brain areas, as determined by Western blot (top) with the 3C11 antibody and ELISA (bottom) using mAb#9 and mAB#1. P21 male WT C57Bl6/J (white bars) mice were compared with male *NFL-Cre BDNF^fl/ko^* mice (black bars) with *bdnf* gene recombination in most pyramidal neurons. Data are mean ± SEM. Hip, Hippocampus; CTX, anterior cortex; STR, striatum; Cereb, cerebellum (*n* = 3 for WT Hip, *n* = 5 for CTX, Cereb, STR, *n* = 2 for *NFL Cre BDNF^fl/ko^* Hip, CTX, STR, Cereb). Raw data are provided in Extended Data Figure 1-1 and Table 2. Image type: ***A***, 2D merged single-plane image; ***B***, ***D***, maximum intensity projection; ***C***, average intensity projection. Scale bars: ***B***, ***D***, 200 µm; ***C***, 100 µm.

BDNF detection is technically challenging because of the low levels of this protein in many brain regions. Relatively highest levels are found in the hippocampus (Ernfors et al., 1990a,b; Hofer et al., 1990; Phillips et al., 1990; Wetmore et al., 1990; Conner et al., 1997; Yan et al., 1997). Using polyclonal antisera, prominent BDNF immunoreactivity is detectable in mossy fiber terminals (Conner et al., 1997; Yan et al., 1997; Zhang et al., 2016). Development of new monoclonal antibodies allowed detection of BDNF in presynaptic terminals of hippocampal mossy fibers and CA3 pyramidal cells (Dieni et al., 2012). In order to optimize detection techniques for BDNF in corticostriatal projection neurons, we first compared different BDNF monoclonal antibodies in sections of mouse hippocampus. As a control for specificity of the staining, conditional BDNF KO mice lacking BDNF mainly in pyramidal neurons through NFL-Cre-mediated recombination (Schweizer et al., 2002) of one allele and full KO of the other allele were used. BDNF immunoreactivity was first determined in hippocampal sections of P21 (Fig. 1*A*) and P56 mice (Fig. 1*B*). Confirming previous studies (Conner et al., 1997; Dieni et al., 2012), we found strong BDNF-IR in mossy fiber terminals (Fig. 1*A*,*B*) using the BDNF mAb#9 antibody (Kolbeck et al., 1999). We then tested additional monoclonal antibodies (clones #4C8, #3C11, #3B2) (Zunino et al., 2016) against BDNF on WT and *NFL-Cre^wt/tg^/BDNF^fl/KO^* hippocampus (Fig. 1*C*). Comparing these antibodies, a strong and highly specific signal was detected with #4C8. Under the same conditions, #3C11 did not show any specific signal, and #3B2 produced relatively high background staining, evident on *NFL-Cre^wt/tg^/BDNF^fl/KO^*-derived hippocampus, which was used as a negative control. As an additional control for the sensitivity of our BDNF staining protocol, we used *BDNF-myc* mice (Matsumoto et al., 2008) and compared BDNF and Myc-IR in the hippocampus. The distribution of the Myc-IR from two independent antibodies and the BDNF signal were virtually identical (Fig. 1*D*). This result confirms the specificity and high sensitivity of our IHC protocol for the detection of endogenous BDNF protein. The staining with mAb#9 and #4C8 was strongly reduced in *NFL-Cre^wt/tg^/BDNF^fl/KO^* hippocampus, correlating with strong reduction of BDNF levels determined by Western blot and ELISA (Fig. 1*E*) (Kolbeck et al., 1999; Rauskolb et al., 2010).

### BDNF levels peak in the cerebral cortex of 3-week-old mice

**Figure 2.**
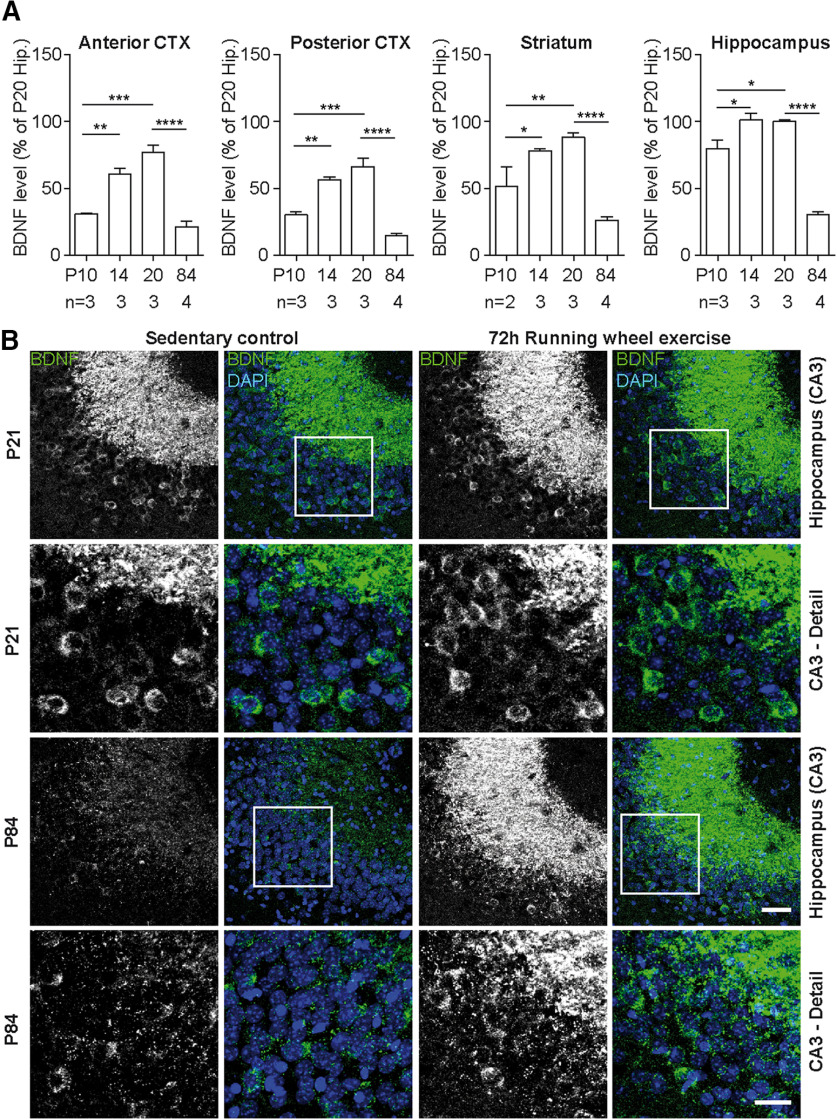
Alterations in brain BDNF levels during postnatal development. ***A***, Sandwich ELISA analysis of relative BDNF protein levels (% of P20 hippocampus) shows a progressive increase of BDNF levels in all analyzed brain areas during the first 3 postnatal weeks. Anterior and posterior cortex shows the relatively highest increase in BDNF expression. Between P20 and P84, BDNF levels are downregulated in cortical and subcortical areas. ***B***, BDNF-IR in hippocampal CA3 area of P21 (rows 1, 2) and P84 (rows 3, 4) mice. Single BDNF-expressing neurons are detected within the pyramidal cell layer in CA3 as well as in mossy fiber terminals at both ages. Voluntary physical activity in a running wheel leads to an increase in hippocampal BDNF-IR, which is more pronounced in 12-week-old animals compared with 3-week-old animals. Statistical analysis: ***A***, One-way ANOVA, Tukey multiple comparison post-test (anterior CTX: *F*_(3,9)_ = 36.18, *p* < 0.0001, ANOVA; posterior CTX: *F*_(3,9)_ = 49.02, *p* < 0.0001, ANOVA; striatum: *F*_(3,8)_ = 34.92, *p* < 0.0001, ANOVA; hippocampus: *F*_(3,9)_ = 78.51; *p* < 0.0001, ANOVA). Data are presented as mean with SEM. *n*, number of independent animals. Raw data are provided in Extended Data Figure 2-1 and Table 2. Image type: ***B***, maximum intensity projection. Scale bar: Hippocampus (CA3), 50 µm; CA3 (Detail), 25 µm. *p < 0.05; **p < 0.01; ***p < 0.001; ****p < 0.0001.

BDNF levels increase during the first 3 weeks of postnatal development (Kolbeck et al., 1999; Gorski et al., 2003; Rauskolb et al., 2010). This increase in BDNF levels during the first 3 postnatal weeks was not only observed in striatum and hippocampus but in particular in anterior and posterior cortex (Fig. 2*A*). We also measured BDNF levels in 12-week-old (P84) mice and found that BDNF levels were significantly lower, by a factor of ∼2-3 in hippocampus and striatum, and a factor of 4-5 in anterior and posterior cortex (Fig. 2*A*). This drop in BDNF protein levels was also reflected by the reduction of BDNF-IR between P21 and P84 hippocampus (Fig. 2*B*, columns 1, 2 from left).

Physical exercise has been reported to stimulate BDNF expression in the CNS of adult rodents (Neeper et al., 1995, 1996; Griesbach et al., 2004; Rasmussen et al., 2009). Exercising in a running wheel for 72 h resulted in a marked increase in hippocampal BDNF-IR, especially in P84 mice (Fig. 2*B*, lanes 3, 4).

### Layer II-III and V corticostriatal neurons express BDNF

**Figure 3.**
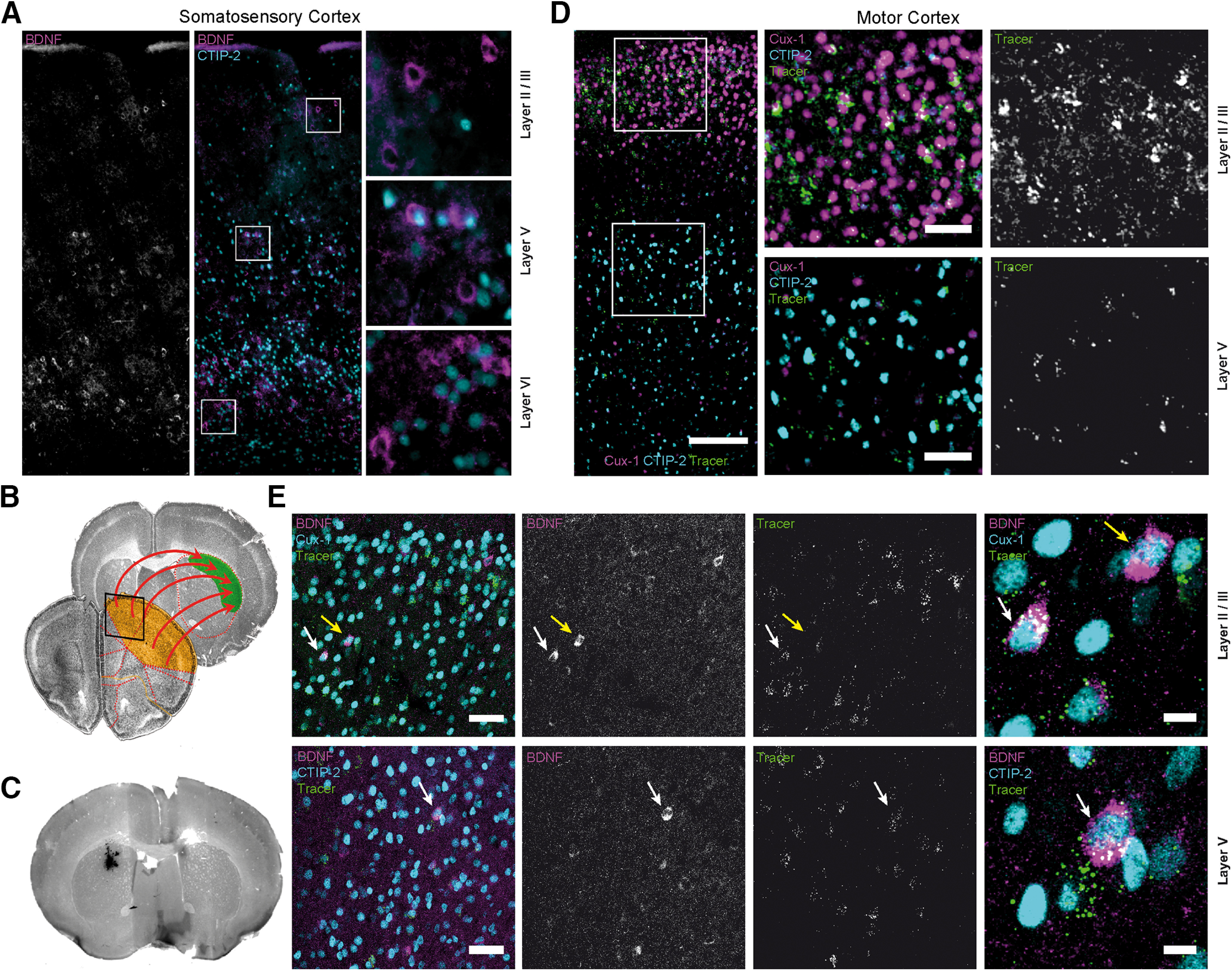
Tracing of corticostriatal projections. ***A***, P21 mouse somatosensory cortex showing BDNF-IR in layers II/III, V, and VI. ***B***, Representative coronal brain sections stained with DAPI. Images represent corticostriatal projections from prefrontal motor cortex (orange, boxed area represents the region depicted in ***D***) to the dorsolateral striatum (green). ***C***, Coronal brain section showing the injection site for fluorescent latex beads in the striatum. ***D***, IHC staining of ipsilateral motor cortex (box in ***B***), which corresponds to the region of highest tracer accumulation within corticostriatal projection neurons. Cux-1 and CTIP-2 label layers II/III and layers V/VI, respectively. Traced neurons were identified in layers II/III (top right) and upper layer V (bottom right). ***E***, BDNF-IR in traced, Cux-1-positive neurons in layer II/III dorsal frontal cortex (top row, white arrow) and CTIP-2-positive neurons in layer V (bottom row, white arrow; see also Extended Data Fig. 3-1*C*,*D*; for statistics, see Extended Data Tables 4-1, 5-1). Not all BDNF-positive neurons contained retrograde tracer beads (yellow arrow). Raw data are provided in Table 2. Image type: ***A–C***, 2D merged single-plane images; ***D***, ***E***, maximum intensity projection. Scale bars: ***D***, Overview, 150 µm; Detail, 50 µm; ***E***, Overview, 50 µm; Detail, 10 µm.

Recently, it has been shown that BDNF in motor cortex plays a role in motor learning (Chen et al., 2019). The motor cortex itself densely innervates the dorsolateral striatum, which is involved in movement control (West et al., 1990). We therefore investigated the types of neurons that express BDNF in these corticostriatal afferents in the third postnatal week when mice acquire adult-like motor skills. Broad expression of BDNF among cortical neurons was reported for rat parietal cortex, showing BDNF-positive pyramidal neurons in layers II/III, V, and VI (Conner et al., 1997). Testing the same cortical area in 3-week-old mice, we found expression of BDNF in the same layers, with highest levels in pyramidal neurons in layers V and VI (Fig. 3*A*) (Conner et al., 1997; Yan et al., 1997). In the motor cortex, corticostriatal projection neurons originate from both layers II/III and V (West et al., 1990). These neurons project to the dorsolateral striatum (Fig. 3*B*). In order to identify these projection neurons from motor cortex, we used fluorescent retrograde tracers that were injected into the dorsolateral part of the striatum (Fig. 3*C*). Traced neurons were enriched in the motor cortex in layers II/III. These neurons also expressed Cux-1 (Fig. 3*D*,*E*), a marker for glutamatergic projection neurons in upper cortical layers (Nieto et al., 2004; Ferrere et al., 2006; Molyneaux et al., 2007). We also detected labeled cells in layer V, which were positive for CTIP-2 (Arlotta et al., 2005; Molyneaux et al., 2007; Jabaudon, 2017) (Fig. 3*D*,*E*). BDNF-IR was found in the somata of individual traced neurons under sedentary conditions in both layers (Fig. 3*E*, white arrows). BDNF expression was only found in a subpopulation of CTIP-2- and Cux-1-positive neurons and only in a subfraction of retrogradely labeled neurons that project to the dorsal striatum (see Extended Data Figs. 4-1, 5-1). This indicates that not all neurons from motor cortex that project to the dorsolateral striatum express BDNF at the same time and that BDNF expression in these neurons could be transiently upregulated in an activity-dependent manner.

BDNF was only detected in a subpopulation (8.5%) of Cux-1-positive neurons in layer II/III and a subpopulation of CTIP-2-positive (9.9%) neurons in layer V (Fig. 3*E*; see Extended Data Figs. 3-1*C*,*D*, 4-1, 5-1). Quantification of BDNF-positive neurons within the group of retrogradely traced corticostriatal afferents revealed that the relative number of traced layer V neurons that also expressed BDNF (27.6%) at P21 (see Extended Data Fig. 5-1) was higher than in layer II/III (13.6%) (see Extended Data Fig. 4-1). At P84, the relative proportion of BDNF-positive projection neurons was lower in both layers (7.9% in layer II/III, 13.1% in layer V). This downregulation suggests that BDNF expression is highest in corticostriatal motor networks in the third postnatal week when mice learn adult-specific motor patterns.

### Mature BDNF and pro-BDNF are both present in cortical projection neurons

The monoclonal mAb#9 antibody (Kolbeck et al., 1999) used in this study detects both mature BDNF and pro-BDNF. For specific detection of pro-BDNF in hippocampus and motor cortex, we used an established rabbit polyclonal antiserum against the BDNF prodomain (see Extended Data Fig. 3-1). We observed pro-BDNF-IR in hippocampal mossy fiber projections and single pyramidal neurons in CA3 (see Extended Data Fig. 3-1*A*), as reported in previous studies using the same pro-BDNF antiserum (Dieni et al., 2012). P28 *NFL-Cre BDNF^fl/ko^*-derived hippocampus revealed a significant reduction of IR for both the pro and mature isoform (see Extended Data Fig. 3-1*B*).

In motor cortex layer II/III (see Extended Data Fig. 3-1*C*) and layer V (see Extended Data Fig. 3-1*D*), pro-BDNF was expressed together with mature BDNF in cell bodies of numerous projection neurons in both layers. However, the expression patterns for mature and pro-BDNF did not fully overlap. Mature BDNF-IR was also detected as a punctuate staining pattern outside of cell bodies, most likely representing afferents in P21 WT cortex. Such signals were much less apparent with the pro-BDNF antiserum. The few stained structures outside cells were hardly distinguishable from background in control *NFL-Cre BDNF^fl/ko^*-derived motor cortex (see Extended Data Fig. 3-1*C*,*D*, right column).

These results indicate that pro-BDNF and mature BDNF IR are detectable in cell bodies of cortical BDNF-expressing neurons. Nevertheless, the expression pattern of both isoforms differs slightly in intensity and spatial distribution. Compared with hippocampus, IR for mature BDNF appears stronger than pro-BDNF in cortical neurons and also is clearly apparent in neurites. However, our data do not allow conclusions about potential functional aspects of pro-BDNF in cortiostriatal-expressing neurons.

### BDNF expression in motor cortex can be increased by physical activity

**Figure 4.**
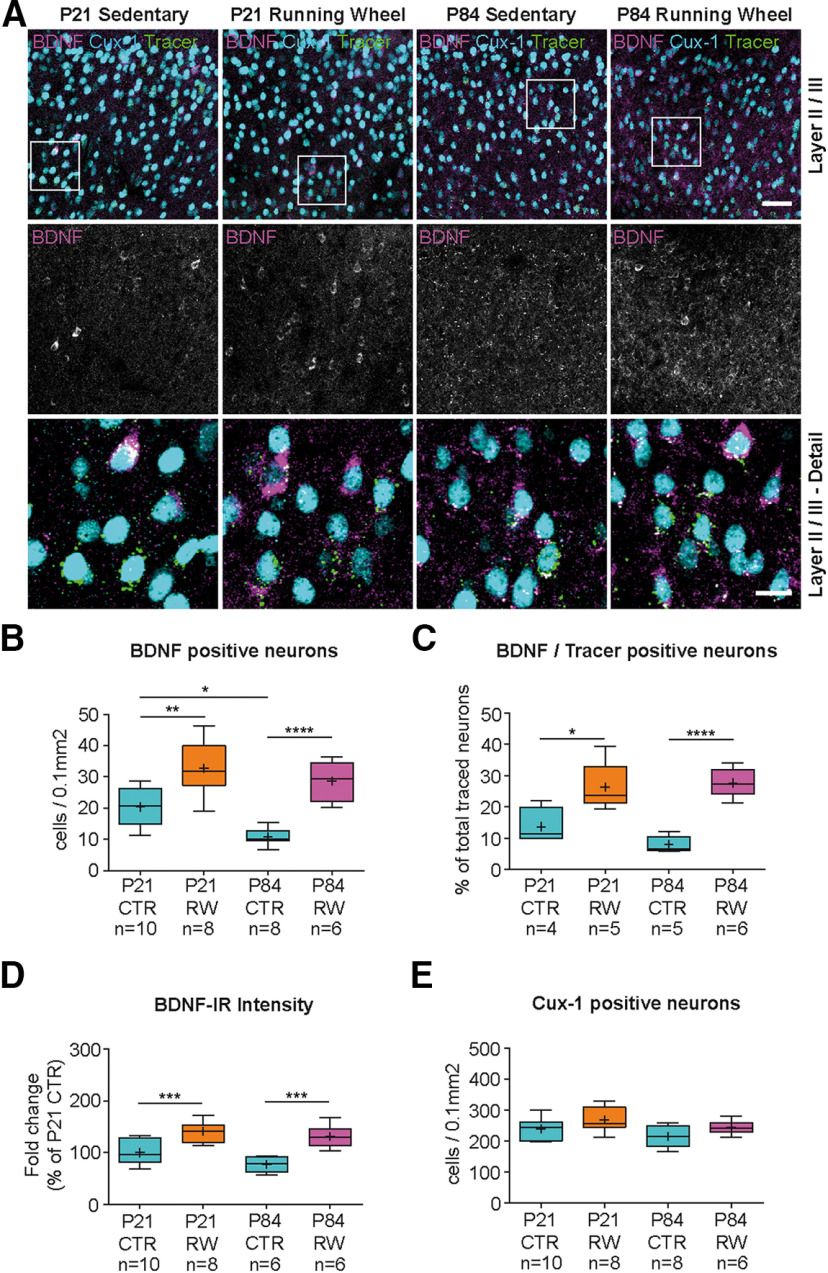
Physical activity increases BDNF expression in layers II/III of the motor cortex. ***A***, BDNF-IR in layers II/III motor cortex (left column in ***A***) shows same image as depicted in Figure 3*E*. P21 sedentary mice (column 1 from left) and runners (column 2); P84 sedentary (column 3) and runners (column 4; see also Extended Data Fig. 4-1). Number of retrogradely traced cortical neurons is shown in Extended Data Figure 4-2. ***B***, Density of BDNF-IR-positive cells in layers II/III dorsal frontal cortex (see also Extended Data Figs. 4-1, 4-3*A*). P84 CTR mice show less BDNF-expressing neurons compared with P21 CTR animals. Physical activity leads to a significant increase in the number of BDNF-IR-positive cells at both ages (see Extended Data Figs. 4-1, 4-4*A-C*). ***C***, BDNF-IR in traced corticostriatal neurons reveals a significant increase in the number of BDNF-positive neurons after physical activity (see Extended Data Fig. 4-1). ***D***, Intensity of BDNF-IR per cell is decreased in layers II/III of P84 compared with P21 mice. Physical activity leads to a significant increase in BDNF-IR per cell at both ages compared with sedentary controls. ***E***, The number of Cux-1-positive, layer II/III neurons and the number of traced neurons (see Extended Data Fig. 4-2*A*) are not affected by age or physical activity. Statistical analysis: one-way ANOVA, Tukey multiple comparison post-test (***B***: *F*_(3,28)_ = 19.21, *p* < 0.0001, ANOVA; ***C***: *F*_(3,16)_ = 16.67, *p* < 0.0001, ANOVA; ***D***: *F*_(3,31)_ = 15.86, *p* < 0.0001, ANOVA; ***E***: *F*_(3,28)_ = 3.290, *p* = 0.0351, ANOVA). Data are presented as box and whiskers (Tukey). +, Mean. Vertical line indicates median. Black dots indicate outliers. *n*, number indicated below. Raw data are provided in Extended Data Figure 4-5 and Table 2. Image type: ***A***, maximum intensity projection. Scale bars: ***A***, 50 µm, Detail, 15 µm. *p < 0.05; **p < 0.01; ***p < 0.001; ****p < 0.0001.

Because cortical BDNF expression peaked around 3 weeks after birth (Fig. 2*A*) when mice learn adult-specific motor patterns, we next tested whether BDNF expression could be modulated by physical activity in Cux-1- and CTIP-2-expressing layer II/III and V corticostriatal neurons. P21 and P84 mice were allowed to exercise in a running wheel for 72 h, and BDNF expression was then analyzed after this training period compared with sedentary controls. In both age groups, mice that exercised showed a high increase in the number of BDNF-positive neurons in layer II/III (Fig. 4*A*,*B*; see Extended Data Figs. 4-1, 4-3*A*, 4-4*A-C*). In the P21 group, running-wheel exercise caused an increase in the overall number of BDNF-positive cells by a factor of 1.6 (Fig. 4*B*; see Extended Data Figs. 4-1, 4-4*A*). In the fraction of traced neurons, this increase was even 1.9-fold (Fig. 4*C*; see Extended Data Fig. 4-1). Interestingly, the adult group at P84 showed even higher effects of physical exercise. We observed a 2.7-fold increase in the total number of BDNF-positive neurons and a 3.5-fold increase in the fraction of traced corticostriatal neurons in layer II/III (Fig. 4*C*; see Extended Data Figs. 4-1, 4-4*B*). This resulted in more BDNF-positive neurons in layer II/III motor cortex of P84 runners than in P21 sedentary mice (Fig. 4*B*; see Extended Data Fig. 4-1). Similarly, we observed an overall increase in BDNF-IR intensity per cell after exercise in layer II/III of P21 and P84 mice (Fig. 4*D*). Exercise did not change the proportion of Cux-1-positive neurons in layer II/III (Fig. 4*E*).

10.1523/JNEUROSCI.0288-20.2020.f1-1 Figure 1-1 Source data of BDNF ELISA depicted in Figure 1:E List of relative BDNF levels measured in cortex, hippocampus, striatum and cerebellum of either P21 C57Bl6/J WT or NFL-Cre BDNF ^fl/ko^ mice, in percent of mean BDNF level in P21 hippocampus. Download Figure 1-1, XLSX file.

10.1523/JNEUROSCI.0288-20.2020.f2-1 Figure 2-1 Source data of BDNF ELISA depicted in Figure 2A**:** List of relative BDNF levels measured in anterior/posterior cortex, hippocampus and striatum of either P10, 14, 20 or P84 C57Bl6/J WT mice, in percent of mean BDNF level in P20 hippocampus. Download Figure 1-1, XLSX file.

10.1523/JNEUROSCI.0288-20.2020.f3-1 Figure 3-1 Detection of BDNF and pro-BDNF in mouse hippocampus and layer II/III & V motor cortex: *A*) BDNF and pro-BDNF-IR detected with mAb#9 antibody and a rabbit polyclonal antiserum against the BDNF pro-domain (ANT-006) in P21 WT C57Bl6/J hippocampus. Pro- and mature BDNF are expressed in mossy fiber projections and single CA3 pyramidal neurons. ***B*)** BDNF and pro-BDNF-IR in P28 NFL-Cre BDNF^fl/ko^ derived hippocampus shows absence of mature BDNF-IR and weak pro-BDNF-IR background noise. **C-*D*)** BDNF and pro-BDNF-IR in layer II/III **(*C*)** and layer V **(*D*)** motor cortex from P21 sedentary control or P28 NFL-Cre BDNF^fl/ko^ mice. Both isoforms are detectable in cell bodies of cortical projection neurons in WT, while immunoreactivity for both antibodies is drastically reduced in NFL-Cre BDNF^fl/ko^ motor cortex. Raw data are provided in **Table 2 - Transparent Reporting**. Image type: maximum intensity projection; Scale bar: ***A***, ***B***) 150 µm, ***C***, ***D***) 50 µm overview; 15 µm detail image. Download Figure 3-1, TIF file.

10.1523/JNEUROSCI.0288-20.2020.f4-1 Figure 4-1 Summary of BDNF expression in layer II/III motor cortex at P21 and P84 sedentary and exercising mice: A-*B*) I and III represent mean values depicted in Figure 4, ***B***, ***C***). **A-*B*)** II represents BDNF expression quantification in layer specific neurons. Download Figure 4-1, XLSX file.

10.1523/JNEUROSCI.0288-20.2020.f4-2 Figure 4-2 Variance of traced neurons in motor cortex of different groups of mice: *A*, *B*) Quantification of tracer positive neurons per area in layers II/III and V motor cortex reveals no significant difference between the four groups. ***C*)** CTIP-2 expression in layer VI of somatosensory cortex is not altered by physical activity or age. Statistical analysis: **A-*C*)** One-way ANOVA, Tukey multiple comparison post-test (A: F(3, 16)=0.5457, *p* = 0.6581, ANOVA; B: F(3, 16)=1.490, *p* = 0.2552, ANOVA; C: F(3, 32)=0.4924, *p* = 0.6901, ANOVA). Data are presented as box and whiskers (Tukey), “+” indicates mean, vertical line median, outliers shown as black dots; n number indicates the number of independent animals used for the analysis. Raw data are provided in **Figure 4-6 - Source data** and **Table 2 - Transparent Reporting**. Download Figure 4-2, TIF file.

10.1523/JNEUROSCI.0288-20.2020.f4-3 Figure 4-3 Cortical layer-specific alterations in BDNF protein and mRNA levels after physical exercise and conditional BDNF ablation: BDNF-IR in layers II/III **(*A*)** and layer V **(*B*)** motor cortex. P21 sedentary mice (left column), 72 h voluntary running-wheel exercise (middle column) and NFL-Cre BDNF^fl/ko^ mice (right column). ***C*)** Images show a representative toluidine blue-stained coronal brain section of motor cortex, used for LMD of layers II/III and V for qRT-PCR analysis. qRT-PCR for BDNF, normalized to GAPDH revealed a reduction of BDNF mRNA by ∼60% in layer II-III and ∼80% in layer V motor cortex in NFL-Cre BDNF^fl/ko^ mice. Data are presented as levels relative to wild-type controls (CTR) in bar graphs for representative visualization of BDNF mRNA reduction (no statistical test was used, because of low n-number); n number indicated below. Raw data are provided in **Figure 4-7 - Source data** and **Table 2 - Transparent Reporting**. Image type: ***A***, ***B***) maximum intensity projection; Scale bar: ***A***, ***B***) 50 µm; 15 µm (detail). Download Figure 4-3, TIF file.

10.1523/JNEUROSCI.0288-20.2020.f4-4 Figure 4-4 Quantification of BDNF expression in cortical neurons by different experimenters and automatic quantification using ImageJ: *A*, *B*) Quantification of BDNF-expressing neurons in P21 **(*A*)** or P84 **(*B*)** cortex using ImageJ versus manual counting by non-blinded investigator (n number indicated below). **C-*E*)** Correlation analysis between the non-blinded investigator and 4 blinded experts for BDNF-positive cell counts in layers II/III **(*C*)**, V **(*D*)** motor cortex and layer VI somatosensory cortex **(*E*)**. 3 random sample images were analyzed for each of the following conditions: P21 CTR, P21 RW, P84 CTR, P84 RW. Statistical analysis: ***A***, ***B***) unpaired *t* test between corresponding pairs (A: LII/III: *t* = 2.147, *p* = 0.0475, investigator: *t* = 3.630, *p* = 0.0023, LV: *t* = 0.8230, *p* = 0.4226, investigator: *t* = 2.487, *p* = 0.0243; LVI: *t* = 0.3224, *p* = 0.7511, investigator: *t* = 0.2480, *p* = 0.8071; B: LII/III: *t* = 3.598, *p* = 0.0037, investigator: *t* = 7.212, *p* < 0.0001, LV: *t* = 2.559, *p* = 0.0251, investigator: *t* = 3.892, *p* = 0.0021; LVI: *t* = 0.9821, *p* = 0.3416), P84 somatosensory CTX layer VI of non-blinded investigator - Mann–Whitney test (Mann–Whitney U 34.00, *p* = 0.8619). C-***E***) Linear regression and correlation analysis, Pearson's R value for correlation is indicated in each graph. Data are presented as box and whiskers (Tukey), “+” indicates mean, vertical line median, outliers shown as black dots; n number indicates the number of independent animals used for the analysis. Raw data are provided in **Figure 4-8 - Source data** and **Table 2 - Transparent Reporting**. Download Figure 4-4, TIF file.

**Figure 5.**
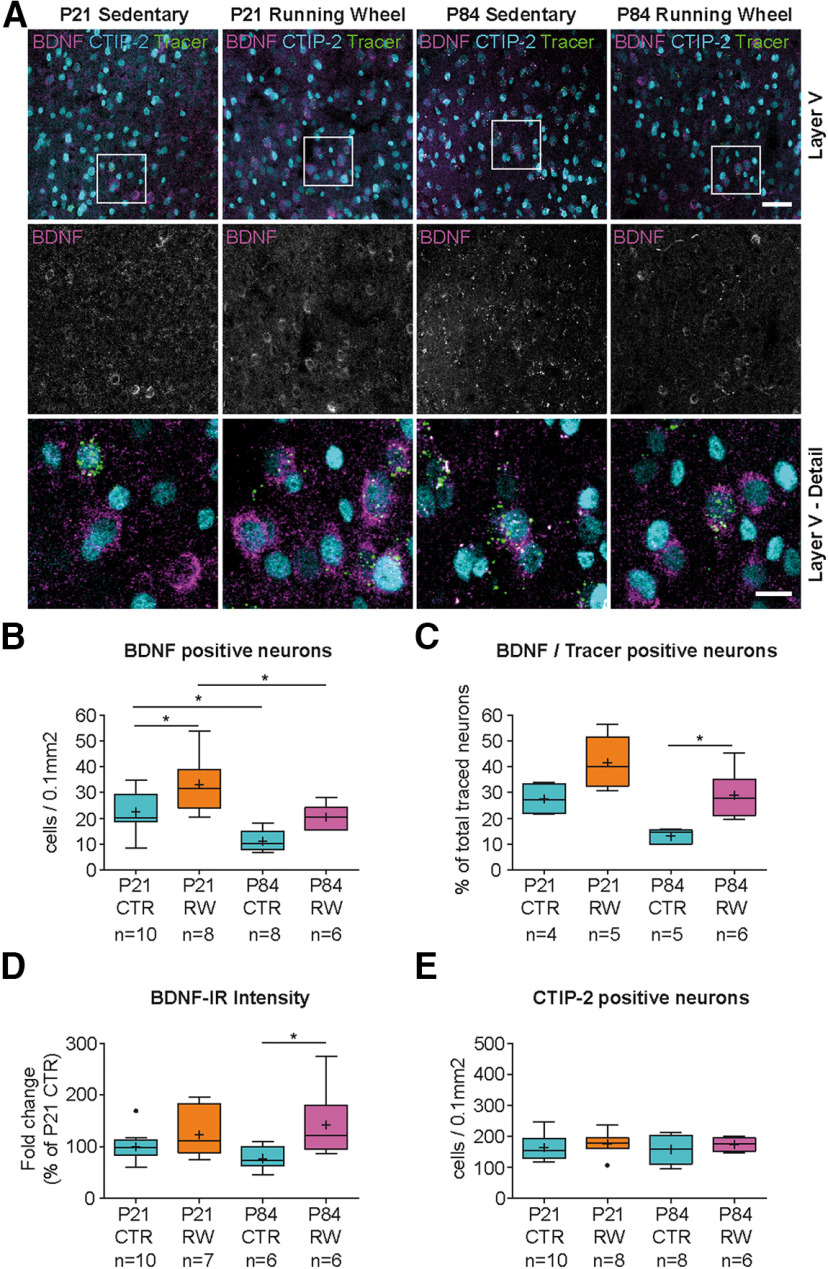
Physical activity leads to minor changes in BDNF expression in layer V motor cortex. ***A***, BDNF-IR in layer V motor cortex. P21 sedentary mice (column 1 from left) and runners (column 2; see also Extended Data Fig. 4-3*B*); P84 sedentary (column 3) and runners (column 4). ***B***, Density of BDNF-IR-positive cells in layer V dorsal frontal cortex (see also Extended Data Figs. 4-4*A*,*B*,*D*, 5-1). P84 CTR mice show less BDNF-expressing neurons compared with P21 CTR animals. At P21, significant differences were only observed when high numbers of sections were analyzed (*n* = 45), whereas no significant increase was observed in the P84 group. ***C***, BDNF-IR in traced corticostriatal neurons reveals a significant increase in the number of BDNF-expressing neurons after physical activity only in the P84 group (see Extended Data Fig. 5-1). ***D***, Physical activity leads to a significant increase in the intensity of BDNF-IR per cell at P84, but not at P21, compared with sedentary controls. ***E***, The number of CTIP-2-positive, layer V neurons and the number of traced neurons (see Extended Data Fig. 4-2*B*) are not affected by age or physical activity. Statistical analysis: one-way ANOVA, Tukey multiple comparison post-test (***B***: *F*_(3,28)_ = 11.69, *p* < 0.0001, ANOVA; ***C***: *F*_(3,16)_ = 11.01, *p* = 0.0004, ANOVA; ***D***: *F*_(3,31)_ = 3.474, *p* = 0.0277, ANOVA; ***E***: *F*_(3,28)_ = 0.3743, *p* = 0.7722, ANOVA). Data are presented as box and whiskers (Tukey). +, Mean. Vertical line indicates median. Black dots indicate outliers. *n*, number indicated below. Raw data are provided in Extended Data Figure 5-2 and Table 2. Image type: ***A***, maximum intensity projection. Scale bars: ***A***, 50 µm, Detail, 15 µm. *p < 0.05.

10.1523/JNEUROSCI.0288-20.2020.f4-5 Figure 4-5 Source data of Figure 4B**-E – Quantification of BDNF and Cux-1 expression in Layer II/III motor cortex: *B*)** List of the number of BDNF-positive neurons per 0.1mm^2^ in P21 and P84 sedentary controls and runner mice (72 h voluntary running-wheel exercise). ***C*)** List of the number of BDNF-positive, traced corticostriatal neurons in percent of the total number of traced neurons in P21 and P84 sedentary controls and runners. ***D*)** List of the intensity of BDNF-IR per cell in P21 and P84 sedentary controls and runners, indicated as fold change in percent of P21 sedentary controls. ***E*)** List of the number of Cux-1-positive in P21 and P84 sedentary controls and runners. Download Figure 4-5, XLSX file.

**Figure 6.**
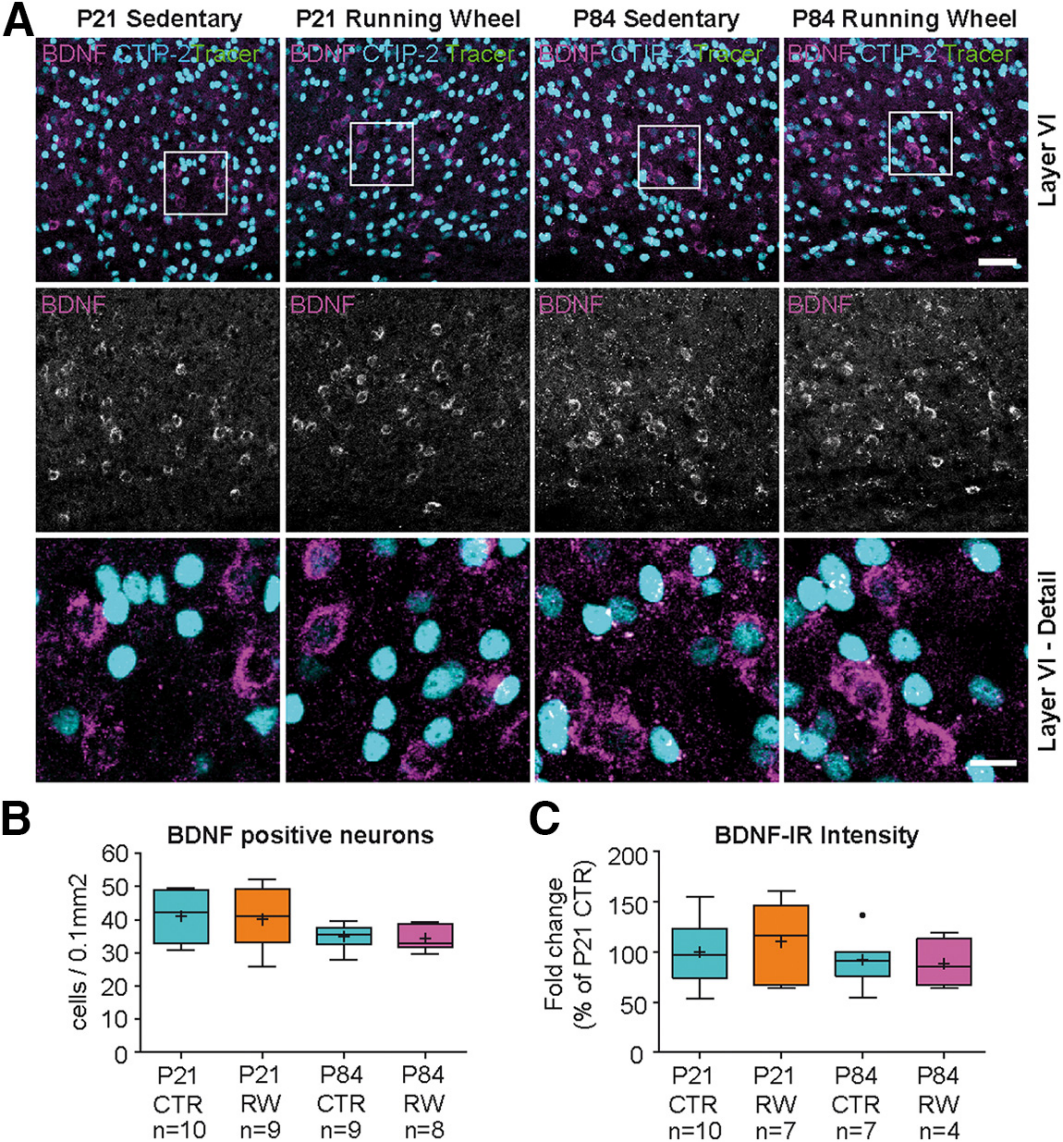
BDNF expression in somatosensory cortex layer VI during postnatal development and after physical activity. ***A***, BDNF-IR in layer VI somatosensory cortex compared in sedentary and exercised animals at P21 (column 1, 2 from left in ***A***) and P84 (column 3, 4 in ***A***). ***B***, Relative number of BDNF-expressing cells per area in somatosensory cortex layer VI is not changed by physical exercise in a running wheel at P21 or P84. ***C***, Quantification of BDNF-IR intensity per cell reveals no significant effect of age or physical activity. The number of CTIP-2-positive, layer VI neurons is not affected by age or physical activity (see Extended Data Fig. 4-2*C*). Statistical analysis: one-way ANOVA, Tukey multiple comparison post-test (***B***: *F*_(3,32)_ = 2.531, *p* = 0.0746, ANOVA; ***C***: *F*_(3,24)_ = 0.5629, *p* = 0.6447, ANOVA). Data are presented as box and whiskers (Tukey). +, Mean. Vertical line indicates median. Black dots indicate outliers. *n*, number indicated below. Raw data are provided in Extended Data Figure 6-1 and Table 2. Scale bars: ***A***, 50 µm, Detail, 15 µm.

10.1523/JNEUROSCI.0288-20.2020.f4-6 Figure 4-6 Source data of Figure 4**-2:** List of the number of traced neurons per 0.1mm^2^ in layer II/III **(*A*)** and layer V **(*B*)** motor cortex of P21 and P84 sedentary controls and runner mice (72 h voluntary running-wheel exercise). ***C*)** List of the number of CTIP-2-positive neurons per 0.1mm^2^ in layer VI somatosensory cortex of P21 and P84 sedentary controls and runners. Download Figure 4-6, XLSX file.

**Figure 7.**
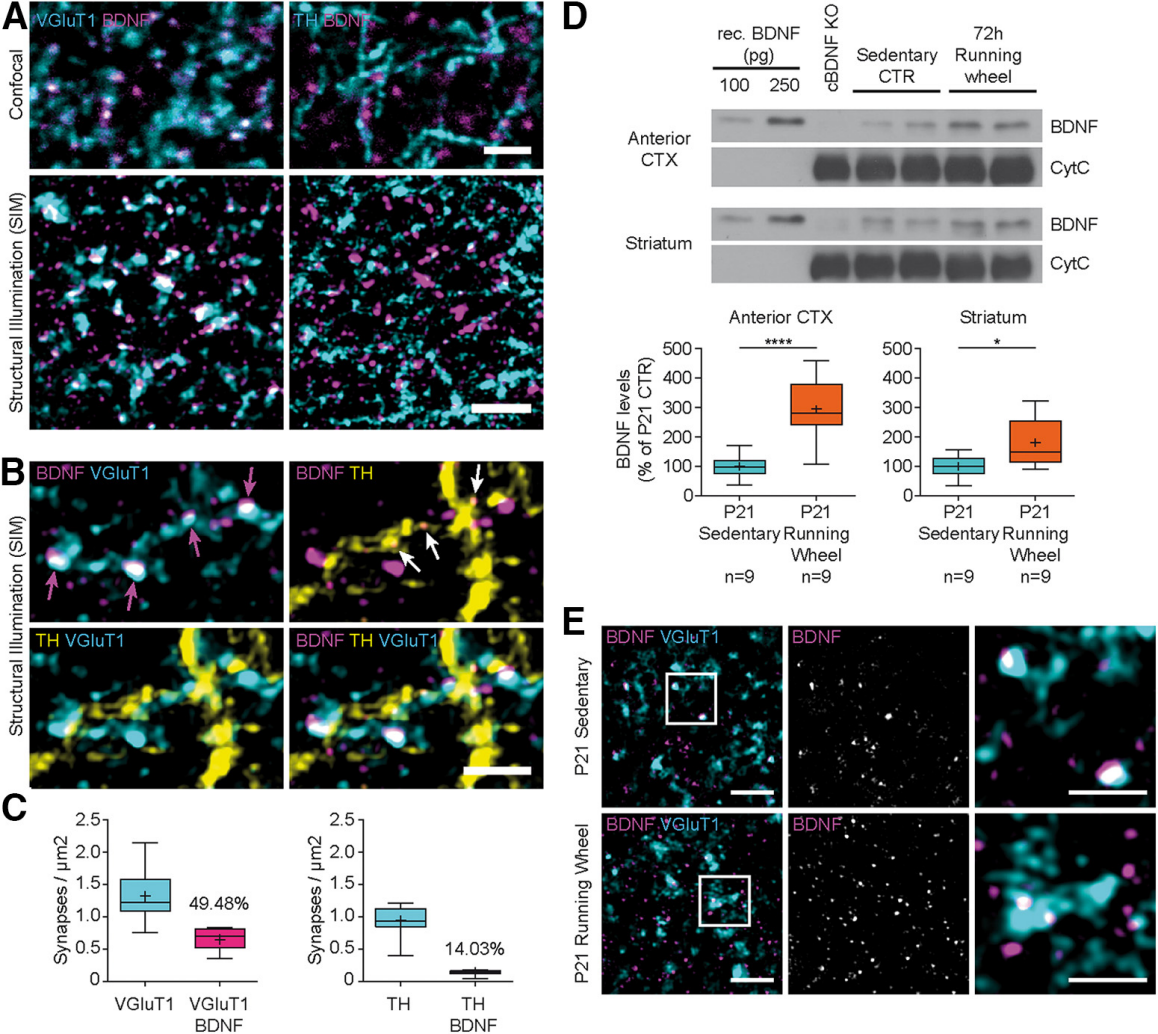
BDNF is enriched in glutamatergic corticostriatal presynaptic terminals. ***A***, Confocal (top) and SIM (bottom) microscopic images showing BDNF-IR in the same section in glutamatergic (left) versus dopaminergic terminals (right) in the dorsal striatum. ***B***, BDNF-IR is present in VGluT1-positive terminals (magenta arrows). Single BDNF-IR signals overlap with TH (white arrows). VGluT1- and TH-positive terminals reside in direct regional proximity but do not overlap. ***C***, Quantification of BDNF signals in VGluT1-positive terminals and TH-positive terminals. True colocalization between BDNF/VGluT1 was confirmed by Costes *p* value (Costes *p* > 0.95) but not between BDNF/TH (Costes *p* ≪ 0.95). ***D***, Representative Western blots showing recombinant BDNF (lanes 1, 2) versus endogenous BDNF derived from anterior cortex or striatum of P21 *NFL-Cre BDNF^fl/ko^* mice (lane 3), P21 sedentary mice (lanes 4, 5), and P21 runners after 72 h voluntary running-wheel exercise (lanes 6, 7); 30 µg of protein lysate was loaded for each sample. BDNF levels were normalized to cytochrome C. Band intensities were determined from extracts of 9 independent mice and presented in % of P21 sedentary mice. Statistical analysis reveals significant increase in BDNF protein levels in both brain areas after running-wheel exercise. Statistical analysis: unpaired *t* test (anterior CTX: *t* = 5,312, *p* < 0.0001; striatum: *t* = 2,784, *p* = 0.0133). ***E***, SIM images showing BDNF-IR in VGluT1-positive terminals in the dorsal striatum in sedentary mice (top row) and after 72 h of voluntary running-wheel exercise (bottom row). Data are presented as box and whiskers (Tukey). +, Mean. Vertical line indicates median. Black dots indicate outliers. *n*, number indicated below. Raw data are provided in Extended Data Figure 7-1 and Table 2. Scale bars: ***A***, 2.5 µm; ***B***, 1.5 µm; ***E***, Overview, 2 µm; Detail, 1 µm. *p < 0.05; ****p < 0.0001.

10.1523/JNEUROSCI.0288-20.2020.f4-7 Figure 4-7 Source data of Figure 4**-3C:** List of relative BDNF mRNA levels in microdissected layer II/III or layer V motor cortex, derived from P21 sedentary C57Bl6/J WT or NFL-Cre BDNF ^fl/ko^ mice in percent of P21 sedentary control (top row). Crossing points obtained from qRT-PCR for BDNF (middle row) or GAPDH (bottom row) are indicated. Download Figure 4-7, XLSX file.

**Figure 8.**
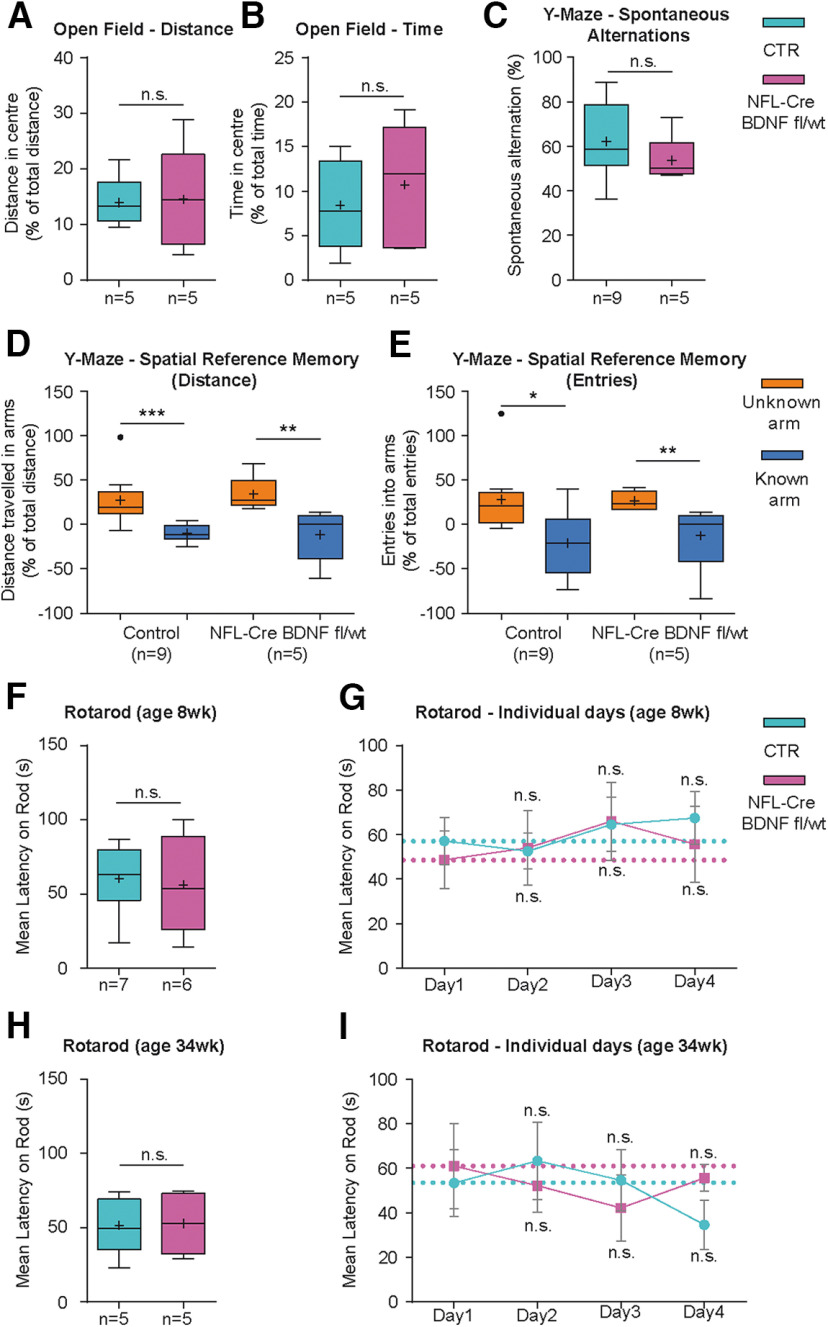
Behavioral analysis of *NFL-Cre BDNF^fl/wt^* mice. ***A***, ***B***, Open Field test: Quantification of distance traveled (***A***) or time spent (***B***) in center (*n*, number indicated below). ***C***, Y-Maze test: Quantification of spontaneous alternations between WT and *NFL-Cre BDNF^fl/wt^* mice during an 8 min run. ***D***, ***E***, Y-Maze test: Quantification of spatial reference memory by analysis of distance traveled (***D***) and number of arm entries (***E***) into unknown versus known arm during the first minute after entering the maze (individual tracks depicted in Extended Data Fig. 8-1). ***F***, ***G***, Rotarod test with 8-week-old mice: Mean latency spent on rod comparing BDNF wt with *NFL-Cre BDNF^fl/wt^* mice (***F***). Mean latency spent on rod on individual days comparing BDNF wt with *NFL-Cre BDNF^fl/wt^* mice (***G***). ***H***, ***I***, Rotarod test with 34-week-old mice. Mean latency spent on rod comparing BDNF wt with *NFL-Cre BDNF^fl/wt^* mice (***H***). Mean latency spent on rod on individual days comparing BDNF wt with *NFL-Cre BDNF^fl/wt^* mice (***I***). *n*, number indicated below. Data are presented as box and whiskers (Tukey). +, Mean. Vertical line indicates median. Black dots indicate outliers (***A–F***,***H***) or mean ± SEM (***G***,***I***). Statistical analysis: ***A***, ***B***, unpaired *t* test (***A***: *t* = 0.1225, *p* = 0.9055, unpaired *t* test; ***B***: *t* = 0.5981, *p* = 0.5663, unpaired *t* test). ***C***, Mann–Whitney test (Mann–Whitney *U* = 11.000, *p* = 0.1454). ***D***, ***E***, Unpaired *t* test (***D***: CTR *t* = 3.559, *p* = 0.0026; *NFL-Cre BDNF^fl/wt^ t* = 2.873, *p* = 0.0207; ***E***: CTR *t* = 2.718, *p* = 0.0152; *NFL-Cre BDNF^fl/wt^ t* = 2.111, *p* = 0.0678). ***F***, ***H***, Unpaired *t* test (***F***: *t* = 0.2778, *p* = 0.7863; ***H***: 0.09904, *p* = 0.9235). ***G***, Two-way ANOVA, Friedman test (non-normal data distribution) revealed no significant improvement in rotarod test comparing day 1 with any of the following days within each group (Friedman statistic CTR: 5.957, *p* = 0.1137; *NFL-Cre BDNF^fl/wt^* 1.696, *p* = 0.6798). One-way ANOVA, Kruskal–Wallis test revealed no difference between WT and *NFL-Cre BDNF^fl/wt^* mice on any of the days tested (Kruskal–Wallis statistic 1.592, *p* = 0.9790). ***I***, One-way ANOVA, Tukey test (normal data distribution WT *F*_(2.264,9.057)_ = 0.8161, *p* = 0.4861, ANOVA) or Friedman test (non-normal distribution *NFL-Cre BDNF^fl/wt^* Friedman statistic: 1.938, *p* = 0.6255) revealed no significant improvement in rotarod test comparing day 1 with any of the following days within each group. One-way ANOVA, Kruskal–Wallis test revealed no difference between WT and *NFL-Cre BDNF^fl/wt^* mice on any of the days tested (Kruskal–Wallis statistic 3.119, *p* = 0.8738). Raw data are provided in Extended Data Figure 8-2 and Table 2. *p < 0.05; **p < 0.01; ***p < 0.001.

10.1523/JNEUROSCI.0288-20.2020.f4-8 Figure 4-8 Source data of Figure 4**-4:** List of the number of BDNF-positive neurons per 0.1mm^2^ in layers II/III and V motor cortex and layer VI somatosensory cortex in either P21 **(*A*)** or P84 **(*B*)** sedentary controls and runner mice obtained by either automatic analysis using Image J or by non-blinded investigator. List of the number of counted BDNF-positive neurons in layer II/III **(*C*)** and V **(*D*)** motor cortex and layer VI somatosensory cortex **(*E*)** (mean value of n = 3 random samples) between non-blinded investigator and four blinded experts. Download Figure 4-8, XLSX file.

10.1523/JNEUROSCI.0288-20.2020.f5-1 Figure 5-1 Summary of BDNF expression in layer V motor cortex at P21 and P84 sedentary and exercising mice: A-*B*) I and III represent mean values depicted in Figure 5, ***B***, ***C***). **A-*B*)** II represents BDNF expression quantification in layer specific neurons. Download Figure 5-1, XLSX file.

10.1523/JNEUROSCI.0288-20.2020.f5-2 Figure 5-2 Source data of Figure 5B**-E – Quantification of BDNF and CTIP-2 expression in Layer V motor cortex: *B*)** List of the number of BDNF-positive neurons per 0.1mm^2^ in P21 and P84 sedentary controls and runner mice (72 h voluntary running-wheel exercise). ***C*)** List of the number of BDNF-positive, traced corticostriatal neurons in percent of the total number of traced neurons in P21 and P84 sedentary controls and runners. ***D*)** List of the intensity of BDNF-IR per cell in P21 and P84 sedentary controls and runners, indicated as fold change in percent of P21 sedentary controls. ***E*)** List of the number of CTIP-2-positive in P21 and P84 sedentary controls and runners. Download Figure 5-2, XLSX file.

10.1523/JNEUROSCI.0288-20.2020.f6-1 Figure 6-1 Source data of Figure 6B, **C – Quantification of BDNF expression in Layer VI somatosensory cortex: *B*)** List of the number of BDNF-positive neurons per 0.1mm^2^ in P21 and P84 sedentary controls and runner mice (72 h voluntary running-wheel exercise). ***C*)** List of the intensity of BDNF-IR per cell in P21 and P84 sedentary controls and runners, indicated as fold change in percent of P21 sedentary controls. Download Figure 6-1, XLSX file.

10.1523/JNEUROSCI.0288-20.2020.f7-1 Figure 7-1 Source data of Figure 7C, D: *C*) List of the raw data used for quantification of BDNF signals in VGluT1 and TH-positive terminals in the striatum. Indicated is the total number and density of counted VGluT1 or TH-positive synapses per µm^2^, the number and density of VGluT1 or TH-positive synapses with BDNF-IR and the amount of BDNF/VGluT1 or BDNF/TH double positive synapses in percent of either all VGluT1 or TH-positive synapses. Costes P value was calculated with ImageJ and is also indicated. ***D*)** List of band intensities of BDNF and cytochrome C, obtained from western blot photograph films using ImageJ. Indicated are the values of anterior cortex and striatum of P21 sedentary controls and runners. In addition, values measured in control tissue, derived from NFL-Cre BDNF ^fl/ko^ mice are listed (not shown in Figure 7). BDNF levels were normalized to cytochrome C and indicated as percent of either P21 sedentary control derived anterior cortex or striatum. Download Figure 7-1, XLSX file.

10.1523/JNEUROSCI.0288-20.2020.f8-1 Figure 8-1 Y-Maze track records during the first minute after opening of the closed arm in the spatial reference memory test run: Documentation of tracks for control mice **(*A*)** and NFL-Cre BDNF^fl/wt^ mice **(*B*)**. Animals were placed in the start-arm (top) and were able to choose between the known arm (blue) and the unknown arm (orange). Download Figure 8-1, TIF file.

10.1523/JNEUROSCI.0288-20.2020.f8-2 Figure 8-2 Sou**rce data of** Figure 8**: *A*)** List of values for distance traveled in center of an open field in percent of total distance comparing C57Bl6/J WT and NFL-Cre BDNF ^fl/wt^ mice. ***B*)** List of values for time spent in center of an open field in percent of total time. ***C*)** List of values for spontaneous alternations in a Y-Maze task with all arms open. ***D*)** List of values for distance traveled in known versus unknown arm in percent of total distance traveled within the first minute after entering a Y-Maze in a spatial reference memory task. ***E*)** Number of arm entries into known versus unknown arm in percent of total arm entries within the first minute after entering a Y-Maze in a spatial reference memory task. List of values for mean latency on a rotarod in seconds obtained from 8 week **(*F*)** or 34 week **(*H*)** old C57Bl6/J WT and NFL-Cre BDNF ^fl/wt^ mice. List of values for latency on a rotarod showing individual 8 week **(*G*)** or 34 week **(*I*)** old animals on four consecutive days. Download Figure 8-2, XLSX file.

**Figure 9.**
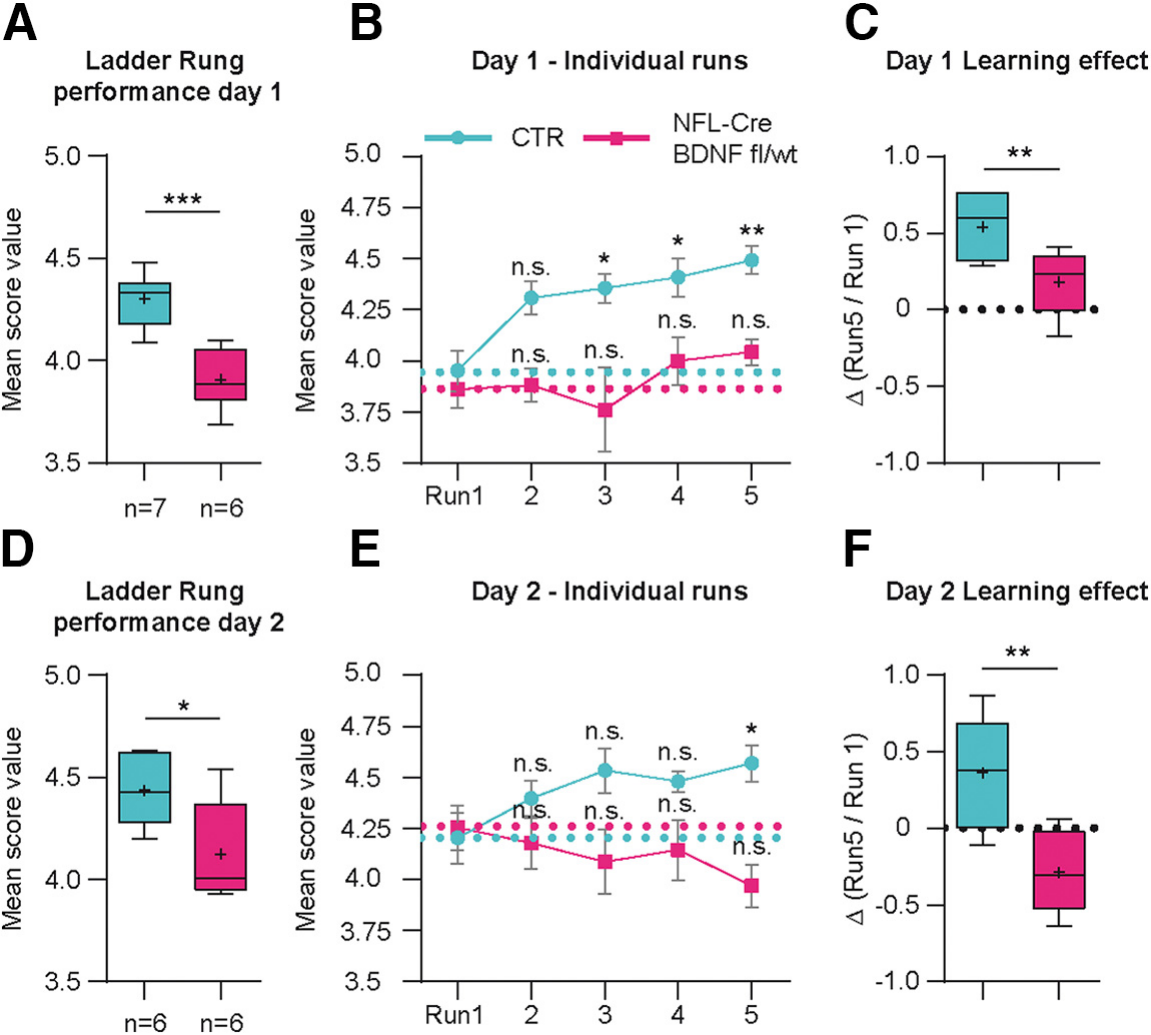
Motor learning is impaired in mice with reduced BDNF in cortical pyramidal neurons. ***A***, ***D***, Mean score values of 3-month-old mice from 5 runs at days 1 and 2 on an irregular ladder rung walking task (unpaired *t* test, ***A***: *t* = 5.170, *p* = 0.0003; Mann–Whitney test, ***D***: Mann–Whitney *U* 5.500, *p* = 0.0476). ***B***, ***E***, Mean score value of individual runs in the irregular ladder rung walking task on day 1 and 2. Asterisks indicate significant difference in mean score value between the individual run and the first run of the particular day of either CTR or *NFL-Cre BDNF^fl/wt^* mice. Dashed lines indicate mean score value of first run (data points indicated as mean ± SEM). Two-way-ANOVA, Bonferroni's multiple comparison test: ***B***: *F*_(4,44)_ = 1.769, *p* = 0.1522; ANOVA, ***E***: *F*_(4,40)_ = 4.515, *p* = 0.0042; ANOVA. ***C***, ***F***, Learning effect indicated as difference in score value between first and last run on the particular day (unpaired *t* test, ***C***: *t* = 3.174, *p* = 0.0089, ***F***: *t* = 3.536, *p* = 0.0054). Data are presented as box and whiskers (Tukey). +, Mean. Vertical line indicates median. Black dots indicate outliers. *n*, number indicated below. Raw data are provided in Extended Data Figure 9-1 and Table 2. *p < 0.05; **p < 0.01; ***p < 0.001.

10.1523/JNEUROSCI.0288-20.2020.f9-1 Figure 9-1 Source data of Figure 9**:** List of mean score values in an irregular ladder rung walking task on day 1 **(*A*)** or day 2 **(*D*)** comparing C57Bl6/J WT and NFL-Cre BDNF ^fl/wt^ mice. List of individual score values of 5 runs comparing C57Bl6/J WT and NFL-Cre BDNF ^fl/wt^ mice on day 1 **(*B*)** and day 2 **(*E*)**. List of learning effect values calculated as difference in score value between first and last run on day 1 **(*C*)** or day 2 **(*F*)**. Download Figure 9-1, XLSX file.

In layer V, exercise-induced BDNF upregulation was less pronounced but still significant (Fig. 5*A*,*B*; see Extended Data Figs. 4-3*B*, 4-4*A*,*B*,*D*, 5-1). Here we found a 1.5-fold increase in the total number of BDNF-expressing neurons (Fig. 5*B*; see Extended Data Figs. 4-4*A*, 5-1). This increase, however, was not significant in the traced subpopulation at P21 (*p* = 0.0732; Fig. 5*C*; see Extended Data Fig. 5-1). Similarly, the number of BDNF-positive neurons at P84 appeared increased by a factor of 1.9 (Fig. 5*B*; see Extended Data Fig. 5-1), but this increase was also not significant (Fig. 5*B*; one-way ANOVA, *p* = 0.1136). However, in the subpopulation of neurons that were retrogradely traced, the 2.2-fold increase in the number of BDNF-positive neurons was significant (*p* = 0.0197) (Fig. 5*C*; see Extended Data Fig. 5-1). In contrast to layer II/III, we observed significant changes in the BDNF-IR intensity only between sedentary and runners at P84, but not P21 (Fig. 5*D*).

Quantification of CTIP-2-positive neurons in layer V also revealed that the number of layer-specific neurons per se was not altered by age or exercise (Fig. 5*E*). The mean number of traced neurons was also not significantly different in both layers and between the different groups, proving the reproducibility of our surgical injections (see Extended Data Fig. 4-2*A*,*B* andFig. 4-6).

To confirm the specificity of BDNF detection in this semiquantitative IHC approach, we also determined the level of gene recombination in these distinct cortical layers in *NFL-Cre BDNF^fl/ko^* control mice. Therefore, BDNF-IR in P21 sedentary was compared with runner and P28 *NFL-Cre BDNF^fl/ko^* mice (see Extended Data Fig. 4-3). As shown before, 72 h of voluntary running-wheel exercise increases BDNF-IR in both layers (see Extended Data Fig. 4-3*A*,*B*, columns 1, 2). In contrast, BDNF expression is drastically reduced in both cortical layers in *NFL-Cre BDNF^fl/ko^* mice (see Extended Data Fig. 4-3*A*,*B*, column 3). To determine how this reduction of BDNF IR in *NFL-Cre BDNF^fl/ko^* mice correlates on the mRNA level, we performed LMD of layers II/III and V motor cortex and analyzed BDNF gene expression by qRT-PCR (see Extended Data Fig. 4-3*C* and Fig. 4-7). We found that BDNF mRNA levels are reduced by ∼60% in layer II/III and ∼80% in layer V. This finding correlates with Western blot and ELISA analyses, showing that BDNF protein levels are highly reduced in anterior cortex of *NFL-Cre BDNF^fl/ko^* mice compared with sedentary WT controls (Fig. 1*E*; see Fig. 7*D*). Runner mice show a significant increase in cortical BDNF levels (see Fig. 7*D*). This supports our observations with IHC on BDNF induction in cortical neurons after running-wheel exercise.

To validate the reliability of the quantified number of BDNF-positive neurons among these conditions, we performed an automatic calculation using the 3D object counter tool in ImageJ (see Extended Data Fig. 4-4*A,B* and Fig. 4-8). This tool confirmed these observations, although higher counts were obtained for P84-derived tissue (see Extended Data Fig. 4-4*B* and Fig. 4-8). This could in part be due to higher background caused by lipofuscin accumulation in this age group. In addition, 4 independent blinded experts manually counted a sample set of images. The interrater reliability for layers II/III and V motor cortex revealed a high level of correlation (see Extended Data Fig. 4-4*C,D* and Fig. 4-8).

These results demonstrate that BDNF can be reexpressed in adult animals in layer II/III and only to a weaker extent in layer V neurons of the motor cortex by physical activity when baseline BDNF levels are low.

### BDNF expression in somatosensory cortex is not altered by motor activity

To test whether this exercise-mediated effect on BDNF expression is specifically related to motor function, we investigated BDNF expression in cortical areas that are not directly related to motor activity. Previous studies have shown that BDNF mRNA is also expressed at relatively high levels in somatosensory cortex layer VI (Conner et al., 1997; Yan et al., 1997; Gorski et al., 2003). We found a similar intense signal in neurons within deep layer VI (Figs. 3*A*, 6*A*). These neurons send dense afferents into the thalamus and are not involved in control of motor function (Deschenes et al., 1998; Llano and Sherman, 2008; Thomson, 2010; McKenna et al., 2011; Kim et al., 2014). Although slightly reduced at P84, we did not observe significant alterations in the number of BDNF-expressing neurons between P21 and P84 (Fig. 6*A*,*B*). In addition, there was no difference in the number of BDNF-positive neurons between sedentary and runner mice: Δ_CTR-P21/RW-P21_ = −0.93 (−2.27%); Δ_CTR-P84/RW-P84_ = −0.45 (−1.29%) (Fig. 6*A*,*B*). This finding was validated by automatic analysis with ImageJ (see Extended Data Fig. 4-4*A,B* and Fig. 4-8) and 4 fully blinded experts (see Extended Data Fig. 4-4*E* and Fig. 4-8). Furthermore, physical activity in a running wheel had no effect on the intensity of BDNF-IR within individual layer VI neurons (Fig. 6*C*). Similarly, the number of layer-specific CTIP-2-positive neurons was not altered under any condition (see Extended Data Fig. 4-2*C* and Fig. 4-8) in this part of the somatosensory cortex. This finding supports the idea that BDNF is specifically upregulated in corticostriatal neurons when mice learn adult motor patterns or adapt to enhanced motor activity in a running wheel.

### BDNF is enriched in corticostriatal presynaptic terminals

We then sought to identify BDNF immunoreactivity in terminals of projecting neurons within the striatum. Expression of BDNF in corticostriatal projection neurons also implies the presence of BDNF in their afferent presynaptic terminals in the striatum. However, striatal medium spiny projection neurons receive BDNF-expressing afferents both from glutamatergiccortical projection neurons and from dopaminergic midbrain derived afferent inputs (Baquet et al., 2005). For this reason, we used costaining of VGluT1 and TH to distinguish these different afferents. High-resolution SIM allowed us to distinguish BDNF immunoreactivity in these different types of afferents (Fig. 7*A*). We found distinct BDNF-IR in VGluT1-positive terminals (Fig. 7*B*, magenta arrows). Few single BDNF-IR punctae were also observed in TH-positive fibers, but they were less numerous and weaker in intensity (Fig. 7*B*, white arrows). Quantification revealed that ∼50% of all VGluT1-positive terminals contained BDNF, whereas only few (14%) TH-positive terminals were BDNF-positive (Fig. 7*C*). This observation was confirmed by quantitative analysis of signal overlap using the Costes *p* value (Costes et al., 2004) revealing true colocalization between BDNF and VGluT1 (Costes *p* > 0.95). This distribution was also observed by conventional confocal analysis (Fig. 7*A*). Unbiased automatic analyses of these images revealed a colocalization of ∼55% between BDNF and VGluT1 and 13% between BDNF and TH, by Pearson's *R* value. These findings indicate that BDNF is present in at least 50% of cortex-derived afferents and presynaptic terminals in the dorsolateral striatum.

We next tested whether the increase in BDNF expression that we observed in corticostriatal projection neurons after physical exercise is also reflected by increased BDNF protein levels in presynaptic terminals within the striatum. For this reason, we performed Western blot experiments using protein extracts from anterior cortex and striatum of P21 sedentary and runner mice and included *NFL-Cre BDNF^fl/ko^*-derived samples as negative control (Fig. 7*D*). Quantitative analyses of the band intensities from these Western blots revealed a significant increase in BDNF protein levels after 72 h of voluntary running-wheel exercise by ∼3-fold in anterior cortex and 1.8-fold in striatum (Fig. 7*D*). Using IHC and high-resolution SIM, we confirmed that BDNF is predominantly present in single VGluT1-positive presynaptic terminals in P21 sedentary and runner mice (Fig. 7*E*). However, quantification of BDNF-IR from this IHC approach appears subpar and not sufficiently reliable to determine an upregulation of BDNF levels in individual terminals because of variability of these weak BDNF-IR signals between individual animals. (Fig. 7*E*).

### Motor learning is impaired in BDNF-deficient mice

The developmental regulation and upregulation of BDNF expression after physical exercise in motor cortex raise the hypothesis that BDNF modulates motor function. Building on previous evidence for a specific role of the motor cortex in motor skill learning (Kawai et al., 2015; Chen et al., 2019), we hypothesize that this function is mediated by BDNF. NFL-Cre mice were chosen because the NFL promotor is predominantly active in large cortical projection neurons (Schweizer et al., 2002). *NFL-Cre BDNF^fl/wt^* mice in which only one BDNF allele is depleted from principal neurons of the cerebral cortex present at a reduction of BDNF levels by ∼50% (Korte et al., 1995). We did not choose a model with >50% cortical BDNF reduction because mice with complete postnatal depletion of BDNF from the nervous system develop anxiety-like behavior (Rauskolb et al., 2010), hyperactivity, freezing, and a clasping phenotype (Baquet et al., 2004; Strand et al., 2007; Rauskolb et al., 2010) that might otherwise mask an effect on corticostriatal motor skill learning. To exclude the possibility that cortical BDNF depletion via NFL-Cre-mediated recombination has effects on spatial memory and learning that also impact motor learning, we performed two different tests. We first used the Open Field test to investigate whether a reduction of cortical BDNF levels by ∼50% causes increased anxiety alongside elevated motor activity. We found that neither the distance traveled nor the time spent in the center of the open field was significantly different between WT and *NFL-Cre BDNF^fl/wt^* mice (Fig. 8*A*,*B*). Next, we tested whether reduced BDNF levels cause memory impairments that could also affect performance in motor tasks. We used a Y-Maze test for analysis of spatial memory (Fig. 8*C-E*) (Kraeuter et al., 2019). The percentage of spontaneous alternations as a measure for short-term spatial memory was not different between WT and *NFL-Cre BDNF^fl/wt^* mice, and both values were in a range that was reported for WT mice at this age (Lamberty and Gower, 1990) (Fig. 8*C*). When mice were allowed access to a new arm that was blocked during the training phase, both groups were able to identify this unknown arm. Both WT and *NFL-Cre BDNF^fl/wt^* mice showed a significant increase in the distance traveled (Fig. 8*D*) and the number of entries (Fig. 8*E*) into the unknown arm, after training (tracks depicted in Extended Data Fig. 8-1). This indicates that *NFL-Cre BDNF^fl/wt^* mice do not show impaired spatial memory because they can remember the arm that was visited before.

To investigate a specific role of the motor cortex for motor skill learning, we tested the performance of the same mice in a rotarod and an irregular ladder rung walking task. Although both tests are used to analyze motor coordination, the rotarod task is especially sensitive to cerebellar dysfunction (Caston et al., 1995; Lalonde et al., 1995; Shiotsuki et al., 2010). In contrast, the basal ganglia circuit is required for motor skill learning of serial motor sequences on an irregular ladder rung (Hikosaka et al., 1999; Shiotsuki et al., 2010). The accelerating rotarod task is less sensitive for evaluating motor skill learning since the learning curve appears flat and the factor for increase in performance after training for several days is low (Perez and Palmiter, 2005; Shiotsuki et al., 2010). Accordingly, we were unable to observe a significant difference in the mean latency on the rotarod between WT and *NFL-Cre BDNF^fl/wt^* mice during a 4 d test trial and at two different ages (Fig. 8*F–I*). Furthermore, mice of both genotypes and both age groups did not show a significant increase in the latency on the rod when compared with the initial run, during a 4 d test trial. (Fig. 8*G*,*I*). We therefore conclude that cortical BDNF reduction by ∼50% via NFL-mediated CRE activity in postnatal brain does not significantly impair cerebellar motor functions in *NFL-Cre BDNF^fl/wt^* mice.

In contrast to the rotarod, the irregular ladder rung walking task captures the capacity to perform and optimize skilled walking, limb placement, and limb coordination on a changing rung pattern. This locomotor challenge depends on synaptic activity in the dorsolateral striatum (West et al., 1990) and forebrain areas related to motor control (Farr et al., 2006; Metz and Whishaw, 2009). Control animals improve performance on an irregular rung pattern within few trials while animals with impairment of the corticostriatal motor circuit present deficits (Metz and Whishaw, 2009). Each animal performed five runs per day. Each run was scored using a 7 category foot fault scoring (Metz and Whishaw, 2002, 2009). We observed that the overall mean score value of adult mice with reduced cortical BDNF expression was significantly lower compared with WT littermates on 2 consecutive days, indicating that motor learning is impaired (Fig. 9*A*,*D*). Both groups showed a similarly low score value in the very first run (Fig. 9*B*). This indicates that reduced performance in the subsequent runs is not because of a developmental defect in mice with reduced cortical BDNF expression because then lower performance would be expected in the initial run in *NFL-Cre BDNF^fl/wt^* mice. WT animals increased their score on both days in consecutive runs, indicating that motor performance improved. This improvement was significant after the third run on day 1 (Fig. 9*B*) and the final run on day 2 (Fig. 9*E*). *NFL-Cre BDNF^fl/wt^* mice failed to significantly improve in subsequent runs on either day (Fig. 9*B*,*E*). To estimate the capability for motor skill learning, the difference in score value between the first and last run was calculated for each day (Fig. 9*C*,*F*). The capability to increase the score value was significantly higher in WT compared with *NFL-Cre BDNF^fl/wt^* mice (Fig. 9*C*,*F*). In contrast, *NFL-Cre BDNF^fl/wt^* mice even revealed a worse performance after the last run on day 2 (Fig. 9*E*,*F*). These data demonstrate that motor learning is impaired in mice in which one *bdnf* allele is depleted by NFL-mediated recombination in corticostriatal projection neurons.

## Discussion

In rodents, the motor cortex plays a central role in motor skill learning rather than skill execution (Kawai et al., 2015). Communication between motor cortex and the dorsolateral striatum is essential for this motor skill learning (West et al., 1990; Dang et al., 2006; Graybiel and Grafton, 2015; Makino et al., 2016; Chen et al., 2019; Sheng et al., 2019). This implies adaptive changes at glutamatergic corticostriatal synapses that cause alterations in striatal medium spiny projection neuron (MSN) firing patterns (Sheng et al., 2019). We show that BDNF is expressed in a subpopulation of layers II/III and V corticostriatal projection neurons, that its expression is high during motor skill acquisition and underlies adaptive changes during postnatal development. This supports previous studies showing that TrkB phosphorylation is upregulated in motor cortex after electrophysiological stimulation (Fritsch et al., 2010) and that BDNF serum levels increase in humans after physical exercise, both indicating that upregulated BDNF could play a role when the motor cortex is activated (Fritsch et al., 2010; Skriver et al., 2014; Chen et al., 2019). However, the neurons within motor cortex in which BDNF is upregulated during motor learning in early postnatal development or during physical exercise in the adult remained uncharacterized so far. Our data fill this gap, showing, for the first time, that layer II/III and V neurons that project from motor cortex to striatum express BDNF in an activity-dependent manner.

Pyramidal cells in layers II/III of the cerebral cortex are generally thought to send projections to other regions of the cortex. By retrograde tracing from the dorsolateral striatum, we identified a subpopulation of BDNF-expressing neurons in layer II/III of the motor cortex that target striatal neurons. A second population of BDNF-expressing corticostriatal cells is located in layer V. In both groups of traced neurons, BDNF expression peaked at P21 (13.7% of traced neurons in layer II/III, 27.6% in layer V), a time at which mice acquire adult-like motor programs (Maltese et al., 2018). It is conceivable that high cortical BDNF levels are required to support synaptic plasticity during this critical phase of motor learning. In line with this idea, BDNF expression was reduced in traced neurons of both layers at P84. Nonetheless, it is so far largely unknown how BDNF expression in those populations is regulated. Although Neeper et al. (1995, 1996) showed that physical exercise increases cortical BDNF expression, they could not differentiate between distinct cortical layers and neuronal subpopulations in the motor cortex. Our data demonstrate that physical exercise increases BDNF expression in defined corticostriatal neuronal subpopulations of layers II/III and V. Surprisingly, the effect of motor exercise was overall stronger in layer II/III than in layer V projecting neurons, and particularly enhanced in neurons projecting to the dorsolateral striatum. Of note, adult mice demonstrated greater exercise-induced increases in BDNF than juvenile mice. This can be explained by lower baseline BDNF levels in adults (Fig. 2*A*) but also suggests that BDNF-mediated plasticity plays a role in motor learning throughout lifetime. Layer II/III contains movement-related neuronal ensembles, which are shaped during motor learning to perform reproducible spatiotemporal sequences of activity (Huber et al., 2012; Peters et al., 2014). This process involves area-specific reorganization of excitatory synapses on layer II/III (Peters et al., 2014) and layer V neurons (Xu et al., 2009), which receive afferent input from layer II/III, in particular in the motor cortex (Kaneko et al., 1994a,b). Both motor learning and running-wheel exercise might thus shape task-related activity in neuronal ensembles of layer II/III motor cortex, leading to activation of layer V neurons via intracortical connections. In line with this idea, a recent study showed that learning of motor tasks leads to enhanced activity of layer V pyramidal neurons (Biane et al., 2019) and that recurrent projections are involved in remodeling motor circuits. Such recurrent projections are required for motor learning and also target layer II/III neurons. It is not fully resolved whether this occurs through direct connections from layer V neurons in the motor cortex to layer II/III neurons in the same region of the cortex or via projections from area 2 of the somatosensory cortex (Yumiya and Ghez, 1984; Sakamoto et al., 1989; Kaneko et al., 1994a). This suggests that BDNF-mediated plasticity is also involved in cortical reorganization during motor learning. In line with this idea, we observed that running-wheel exercise leads to much higher increase in BDNF protein levels within the anterior cortex compared with striatum (Fig. 7*D*). This indicates that only a part of BDNF is translocated in corticostriatal afferents and that BDNF is also upregulated in corticocortical projections for remodeling intracortical networks. It is also possible that elevated BDNF does not only change synaptic strength but also alters other parameters in motor circuits, such as the level of myelination of projecting fibers via regulation of myelin formation (Vondran et al., 2010; Xiao et al., 2010). Surprisingly, BDNF expression in corticothalamic neurons of layer VI somatosensory cortex was not affected by enhanced motor activity, indicating that physical exercise does not lead to a generalized upregulation of BDNF in cortical pyramidal neurons. Thus, enhanced BDNF expression appears restricted to subpopulations in which increased activity and plasticity are necessary for the relevant adaption to the environment. In conclusion, our data suggest that layer- and pathway-specific BDNF expression supports motor learning during development and that this function is retained throughout adulthood.

The question remains how BDNF acts in the corticostriatal circuit to mediate its effects on motor learning. We identified BDNF in ∼50% of VGluT1-positive presynaptic terminals within the dorsolateral striatum, indicating a central role of BDNF in modulating synaptic strength at these synapses, a basic process in motor learning (Park et al., 2014). BDNF secretion from these terminals putatively requires increased activity in the corticostriatal pathway (Huber et al., 2012; Peters et al., 2014), which is triggered by physical exercise (Neeper et al., 1996). This then could cause presynaptic NMDAR activation and Ca^2+^-mediated BDNF release (Park et al., 2014). As a consequence, similar to neuronal ensembles in the motor cortex (Xu et al., 2009; Huber et al., 2012; Peters et al., 2014), MSNs in the dorsolateral striatum appear to change their firing pattern in a motor skill-specific manner (Sheng et al., 2019). This is accompanied by stabilization of increased firing of D1-MSNs during task performance and increased firing of D2-MSNs during intertrial intervals (Sheng et al., 2019). These findings also suggest that distinct mechanisms modulate BDNF responsiveness in D1 versus D2 neurons. Experiments with conditional KO of TrkB indicate that D1 and D2 MSNs are differentially sensitive to TrkB depletion (Baydyuk et al., 2011). Together, these previous findings and our results indicate that corticostriatal synaptic plasticity is modulated by BDNF. These synapses appear sensitive to relatively mild reduction of BDNF levels in *NFL-Cre BDNF^fl/wt^* mice, much more than neuronal circuits in the hippocampus and cerebellum that are also regulated by BDNF expression. *NFL-Cre BDNF^fl/wt^* mice performed well in an accelerating rotarod task, which is known to be sensitive to cerebellar dysfunction (Caston et al., 1995; Lalonde et al., 1995; Shiotsuki et al., 2010) and less sensitive for motor skill learning (Perez and Palmiter, 2005; Shiotsuki et al., 2010). Impaired motor skill learning in transgenic *NFL-Cre BDNF^fl/wt^* mice was furthermore not a consequence of defective hippocampal function for spatial memory since those mice behaved like control animals in a Y-Maze test.

These data also suggest that BDNF differentially impacts formation and maintenance of memories for spatial tasks and for motor skills. Patients suffering from Alzheimer's disease show normal motor skill learning capabilities while being unable to recognize faces or learn series of words (Eslinger and Damasio, 1986). Similarly, musicians suffering from amnesia or dementia with massive deficits in explicit memory can perfectly play music and do not show a loss of motor skills at the same level as they deteriorate in other cognitive functions (Laforce and Doyon, 2001; Cavaco et al., 2011, 2012; Hsieh et al., 2011; Finke et al., 2012). This has led to the hypothesis that the learning systems for declarative and procedural memories are independent (Eslinger and Damasio, 1986). BDNF-mediated plasticity at corticostriatal neurons thus appears necessary for procedural memory formation and motor skill learning and is independent from the hippocampus and mechanisms of declarative memory formation. Our data show that BDNF has a prominent molecular function for plasticity at corticostriatal synapses, which is in line with the observation that corticostriatal LTP is abolished when BDNF is depleted from motor cortex (Park et al., 2014). However, Park et al. (2014) suspected that theta-burst stimulation mediated BDNF release in the striatum originates from neurons in layer VI motor cortex. Our tracing data reveal striatum-projecting neurons in virtually all layers, yet suggest that the sources of cortical BDNF for motor-learning are primarily located within layers II/III and V.

More globally, BDNF-mediated plasticity during motor learning could shape three distinct synaptic connections originating from the motor cortex. First, BDNF could contribute to intracortical reorganization between layers II/III and V in motor cortex. Second, corticostriatal plasticity appears regulated by BDNF from neurons in layers II/III and V of motor cortex projecting to the dorsolateral striatum. Third, the strength of synapses from striatal collaterals derived from corticospinal projections that originate from layer V motor cortex could be regulated by BDNF. According to this idea, specific motor tasks might shape activity in task-related neuronal ensembles in layer II/III (Peters et al., 2014) and induce BDNF expression. Those layer II/III neurons then innervate intracortical and subcortical targets. Intracortically, they might increase activity and thus induce BDNF expression in layer V neurons (Xu et al., 2009). Our tracing experiments revealed that BDNF-expressing layer II/III and V neurons also directly target the dorsolateral striatum, pointing to BDNF-mediated synaptic plasticity on corticostriatal synapses. This could be important to forward altered modes of cortical activity to striatal MSNs, which undergo changes in synaptic turnover and activity in a motor task-specific manner (Sheng et al., 2019). Finally, the subpopulation of BDNF-expressing neurons in layer V might also project to the spinal cord or govern corticospinal projection neurons (Biane et al., 2019). Those neurons target spinal cord interneurons and send their collaterals to the striatum (Molyneaux et al., 2007). Thus, they also could be marked after tracer injection into the striatum. Our tracing experiments could not distinguish such corticospinal neurons from layer V neurons, which exclusively project to the striatum. However, layer V corticospinal projection neurons represent the main motor output pathway from the basal ganglia circuit, and it appears possible that BDNF in corticospinal projection neurons modulates synaptic plasticity and activity on related synapses in the spinal cord.

In the present study, we focused on motor-related changes in BDNF expression in the corticostriatal system using transgenic *NFL-Cre BDNF^fl/wt^* mice. Future studies are needed to address the role of BDNF-mediated intracortical and corticospinal plasticity related to motor skill learning. However, the alterations in corticostriatal BDNF signaling that are found in various movement disorders, such as Huntington's disease (Plotkin et al., 2014) and dystonia (Maltese et al., 2018) likely constitute important pathophysiological processes of these conditions. A better understanding of the function and regulation of BDNF in different neuronal populations and corresponding synapses in the motor pathway will not only improve our understanding of neurodegenerative diseases. It will also help to develop new therapeutic approaches in clinical situations demanding motor skill learning, such as for motor-impaired patients recovering from stroke.

## References

[B1] Alexander GE, DeLong MR, Strick PL (1986) Parallel organization of functionally segregated circuits linking basal ganglia and cortex. Annu Rev Neurosci 9:357–381. 10.1146/annurev.ne.09.030186.002041 3085570

[B2] Altar CA, Cai N, Bliven T, Juhasz M, Conner JM, Acheson AL, Lindsay RM, Wiegand SJ (1997) Anterograde transport of brain-derived neurotrophic factor and its role in the brain. Nature 389:856–860. 10.1038/39885 9349818

[B3] Arlotta P, Molyneaux BJ, Chen J, Inoue J, Kominami R, Macklis JD (2005) Neuronal subtype-specific genes that control corticospinal motor neuron development in vivo. Neuron 45:207–221. 10.1016/j.neuron.2004.12.036 15664173

[B4] Baquet ZC, Gorski JA, Jones KR (2004) Early striatal dendrite deficits followed by neuron loss with advanced age in the absence of anterograde cortical brain-derived neurotrophic factor. J Neurosci 24:4250–4258. 10.1523/JNEUROSCI.3920-03.2004 15115821 PMC6729276

[B5] Baquet ZC, Bickford PC, Jones KR (2005) Brain-derived neurotrophic factor is required for the establishment of the proper number of dopaminergic neurons in the substantia nigra pars compacta. J Neurosci 25:6251–6259. 10.1523/JNEUROSCI.4601-04.2005 15987955 PMC6725062

[B6] Barde YA, Edgar D, Thoenen H (1982) Purification of a new neurotrophic factor from mammalian brain. EMBO J 1:549–553. 7188352 10.1002/j.1460-2075.1982.tb01207.x PMC553086

[B7] Baydyuk M, Russell T, Liao GY, Zang K, An JJ, Reichardt LF, Xu B (2011) TrkB receptor controls striatal formation by regulating the number of newborn striatal neurons. Proc Natl Acad Sci USA 108:1669–1674. 10.1073/pnas.1004744108 21205893 PMC3029684

[B8] Biane JS, Takashima Y, Scanziani M, Conner JM, Tuszynski MH (2019) Reorganization of recurrent layer 5 corticospinal networks following adult motor training. J Neurosci 39:4684–4693. 10.1523/JNEUROSCI.3442-17.2019 30948479 PMC6561695

[B9] Caston J, Jones N, Stelz T (1995) Role of preoperative and postoperative sensorimotor training on restoration of the equilibrium behavior in adult mice following cerebellectomy. Neurobiol Learn Mem 64:195–202. 10.1006/nlme.1995.0002 8564373

[B10] Cavaco S, Feinstein JS, van Twillert H, Tranel D (2012) Musical memory in a patient with severe anterograde amnesia. J Clin Exp Neuropsychol 34:1089–1100. 10.1080/13803395.2012.728568 23036073 PMC3919540

[B11] Cavaco S, Anderson SW, Correia M, Magalhaes M, Pereira C, Tuna A, Taipa R, Pinto P, Pinto C, Cruz R, Lima AB, Castro-Caldas A, da Silva AM, Damasio H (2011) Task-specific contribution of the human striatum to perceptual-motor skill learning. J Clin Exp Neuropsychol 33:51–62. 10.1080/13803395.2010.493144 20603739

[B12] Chen G, Kolbeck R, Barde YA, Bonhoeffer T, Kossel A (1999) Relative contribution of endogenous neurotrophins in hippocampal long-term potentiation. J Neurosci 19:7983–7990. 10.1523/JNEUROSCI.19-18-07983.1999 10479698 PMC6782442

[B13] Chen K, Zheng Y, Wei JA, Ouyang H, Huang X, Zhang F, Lai CS, Ren C, So KF, Zhang L (2019) Exercise training improves motor skill learning via selective activation of mTOR. Sci Adv 5:eaaw1888. 10.1126/sciadv.aaw1888 31281888 PMC6609215

[B14] Conner JM, Lauterborn JC, Yan Q, Gall CM, Varon S (1997) Distribution of brain-derived neurotrophic factor (BDNF) protein and mRNA in the normal adult rat CNS: evidence for anterograde axonal transport. J Neurosci 17:2295–2313. 10.1523/JNEUROSCI.17-07-02295.1997 9065491 PMC6573520

[B15] Cospito JA, Kultas-Ilinsky K (1981) Synaptic organization of motor corticostriatal projections in the rat. Exp Neurol 72:257–266. 10.1016/0014-4886(81)90221-1 7238688

[B16] Costes SV, Daelemans D, Cho EH, Dobbin Z, Pavlakis G, Lockett S (2004) Automatic and quantitative measurement of protein-protein colocalization in live cells. Biophys J 86:3993–4003. 10.1529/biophysj.103.038422 15189895 PMC1304300

[B17] Dang MT, Yokoi F, Yin HH, Lovinger DM, Wang Y, Li Y (2006) Disrupted motor learning and long-term synaptic plasticity in mice lacking NMDAR1 in the striatum. Proc Natl Acad Sci USA 103:15254–15259. 10.1073/pnas.0601758103 17015831 PMC1622809

[B18] Deschenes M, Veinante P, Zhang ZW (1998) The organization of corticothalamic projections: reciprocity versus parity. Brain Res Brain Res Rev 28:286–308. 10.1016/s0165-0173(98)00017-4 9858751

[B19] Dieni S, Matsumoto T, Dekkers M, Rauskolb S, Ionescu MS, Deogracias R, Gundelfinger ED, Kojima M, Nestel S, Frotscher M, Barde YA (2012) BDNF and its pro-peptide are stored in presynaptic dense core vesicles in brain neurons. J Cell Biol 196:775–788. 10.1083/jcb.201201038 22412021 PMC3308691

[B20] Donoghue JP, Herkenham M (1986) Neostriatal projections from individual cortical fields conform to histochemically distinct striatal compartments in the rat. Brain Res 365:397–403. 10.1016/0006-8993(86)91658-6 3004664

[B21] Edelmann E, Cepeda-Prado E, Franck M, Lichtenecker P, Brigadski T, Leßmann V (2015) Theta burst firing recruits BDNF release and signaling in postsynaptic CA1 neurons in spike-timing-dependent LTP. Neuron 86:1041–1054. 10.1016/j.neuron.2015.04.007 25959732

[B22] Erickson KI, Prakash RS, Voss MW, Chaddock L, Heo S, McLaren M, Pence BD, Martin SA, Vieira VJ, Woods JA, McAuley E, Kramer AF (2010) Brain-derived neurotrophic factor is associated with age-related decline in hippocampal volume. J Neurosci 30:5368–5375. 10.1523/JNEUROSCI.6251-09.2010 20392958 PMC3069644

[B23] Ernfors P, Wetmore C, Olson L, Persson H (1990a) Identification of cells in rat brain and peripheral tissues expressing mRNA for members of the nerve growth factor family. Neuron 5:511–526. 10.1016/0896-6273(90)90090-3 2206535

[B24] Ernfors P, Ibanez CF, Ebendal T, Olson L, Persson H (1990b) Molecular cloning and neurotrophic activities of a protein with structural similarities to nerve growth factor: developmental and topographical expression in the brain. Proc Natl Acad Sci USA 87:5454–5458. 10.1073/pnas.87.14.5454 2164684 PMC54343

[B25] Eslinger PJ, Damasio AR (1986) Preserved motor learning in Alzheimer's disease: implications for anatomy and behavior. J Neurosci 6:3006–3009. 3760945 10.1523/JNEUROSCI.06-10-03006.1986 PMC6568800

[B26] Farr TD, Liu L, Colwell KL, Whishaw IQ, Metz GA (2006) Bilateral alteration in stepping pattern after unilateral motor cortex injury: a new test strategy for analysis of skilled limb movements in neurological mouse models. J Neurosci Methods 153:104–113. 10.1016/j.jneumeth.2005.10.011 16309746

[B27] Ferrere A, Vitalis T, Gingras H, Gaspar P, Cases O (2006) Expression of Cux-1 and Cux-2 in the developing somatosensory cortex of normal and barrel-defective mice. Anat Rec A Discov Mol Cell Evol Biol 288:158–165. 10.1002/ar.a.20284 16419078

[B28] Finke C, Esfahani NE, Ploner CJ (2012) Preservation of musical memory in an amnesic professional cellist. Curr Biol 22:R591–R592. 10.1016/j.cub.2012.05.041 22877775

[B29] Fraser SA, Li KZ, Penhune VB (2009) A comparison of motor skill learning and retention in younger and older adults. Exp Brain Res 195:419–427. 10.1007/s00221-009-1806-5 19404628

[B30] Fritsch B, Reis J, Martinowich K, Schambra HM, Ji Y, Cohen LG, Lu B (2010) Direct current stimulation promotes BDNF-dependent synaptic plasticity: potential implications for motor learning. Neuron 66:198–204. 10.1016/j.neuron.2010.03.035 20434997 PMC2864780

[B31] Gorski JA, Zeiler SR, Tamowski S, Jones KR (2003) Brain-derived neurotrophic factor is required for the maintenance of cortical dendrites. J Neurosci 23:6856–6865. 12890780 10.1523/JNEUROSCI.23-17-06856.2003 PMC6740724

[B32] Graybiel AM, Grafton ST (2015) The striatum: where skills and habits meet. Cold Spring Harb Perspect Biol 7:a021691. 10.1101/cshperspect.a021691 26238359 PMC4526748

[B33] Griesbach GS, Hovda DA, Molteni R, Wu A, Gomez-Pinilla F (2004) Voluntary exercise following traumatic brain injury: brain-derived neurotrophic factor upregulation and recovery of function. Neuroscience 125:129–139. 10.1016/j.neuroscience.2004.01.030 15051152

[B34] Grillner S (2015) Action: the role of motor cortex challenged. Curr Biol 25:R508–R511. 10.1016/j.cub.2015.04.023 26079084

[B35] Hikosaka O, Nakahara H, Rand MK, Sakai K, Lu X, Nakamura K, Miyachi S, Doya K (1999) Parallel neural networks for learning sequential procedures. Trends Neurosci 22:464–471. 10.1016/s0166-2236(99)01439-3 10481194

[B36] Hofer M, Pagliusi SR, Hohn A, Leibrock J, Barde YA (1990) Regional distribution of brain-derived neurotrophic factor mRNA in the adult mouse brain. EMBO J 9:2459–2464. 2369898 10.1002/j.1460-2075.1990.tb07423.x PMC552273

[B37] Hsieh S, Hornberger M, Piguet O, Hodges JR (2011) Neural basis of music knowledge: evidence from the dementias. Brain 134:2523–2534. 10.1093/brain/awr190 21857031

[B38] Huber D, Gutnisky DA, Peron S, O'Connor DH, Wiegert JS, Tian L, Oertner TG, Looger LL, Svoboda K (2012) Multiple dynamic representations in the motor cortex during sensorimotor learning. Nature 484:473–478. 10.1038/nature11039 22538608 PMC4601999

[B39] Jabaudon D (2017) Fate and freedom in developing neocortical circuits. Nat Commun 8:16042. 10.1038/ncomms16042 28671189 PMC5500875

[B40] Kalivas PW (2009) The glutamate homeostasis hypothesis of addiction. Nat Rev Neurosci 10:561–572. 10.1038/nrn2515 19571793

[B41] Kaneko T, Caria MA, Asanuma H (1994a) Information processing within the motor cortex: I. Responses of morphologically identified motor cortical cells to stimulation of the somatosensory cortex. J Comp Neurol 345:161–171. 10.1002/cne.903450202 7929897

[B42] Kaneko T, Caria MA, Asanuma H (1994b) Information processing within the motor cortex: II. Intracortical connections between neurons receiving somatosensory cortical input and motor output neurons of the cortex. J Comp Neurol 345:172–184. 10.1002/cne.903450203 7929898

[B43] Kawai R, Markman T, Poddar R, Ko R, Fantana AL, Dhawale AK, Kampff AR, Olveczky BP (2015) Motor cortex is required for learning but not for executing a motor skill. Neuron 86:800–812. 10.1016/j.neuron.2015.03.024 25892304 PMC5939934

[B44] Kim J, Matney CJ, Blankenship A, Hestrin S, Brown SP (2014) Layer 6 corticothalamic neurons activate a cortical output layer, layer 5a. J Neurosci 34:9656–9664. 10.1523/JNEUROSCI.1325-14.2014 25031405 PMC4099543

[B45] Kimura M, Aosaki T, Ishida A (1993) Neurophysiological aspects of the differential roles of the putamen and caudate nucleus in voluntary movement. Adv Neurol 60:62–70. 8380529

[B46] King BR, Fogel SM, Albouy G, Doyon J (2013) Neural correlates of the age-related changes in motor sequence learning and motor adaptation in older adults. Front Hum Neurosci 7:142. 10.3389/fnhum.2013.00142 23616757 PMC3628357

[B47] Kolbeck R, Bartke I, Eberle W, Barde YA (1999) Brain-derived neurotrophic factor levels in the nervous system of wild-type and neurotrophin gene mutant mice. J Neurochem 72:1930–1938. 10.1046/j.1471-4159.1999.0721930.x 10217270

[B48] Korte M, Carroll P, Wolf E, Brem G, Thoenen H, Bonhoeffer T (1995) Hippocampal long-term potentiation is impaired in mice lacking brain-derived neurotrophic factor. Proc Natl Acad Sci USA 92:8856–8860. 10.1073/pnas.92.19.8856 7568031 PMC41066

[B49] Korte M, Kang H, Bonhoeffer T, Schuman E (1998) A role for BDNF in the late-phase of hippocampal long-term potentiation. Neuropharmacology 37:553–559. 10.1016/s0028-3908(98)00035-5 9704996

[B50] Kraeuter AK, Guest PC, Sarnyai Z (2019) The Y-Maze for assessment of spatial working and reference memory in mice. Methods Mol Biol 1916:105–111. 30535688 10.1007/978-1-4939-8994-2_10

[B51] Kunzle H (1975) Bilateral projections from precentral motor cortex to the putamen and other parts of the basal ganglia: an autoradiographic study in *Macaca fascicularis*. Brain Res 88:195–209. 50112 10.1016/0006-8993(75)90384-4

[B52] Laforce R Jr, Doyon J (2001) Distinct contribution of the striatum and cerebellum to motor learning. Brain Cogn 45:189–211. 10.1006/brcg.2000.1237 11237366

[B53] Lalonde R, Bensoula AN, Filali M (1995) Rotorod sensorimotor learning in cerebellar mutant mice. Neurosci Res 22:423–426. 10.1016/0168-0102(95)00916-h 7478307

[B54] Lamberty Y, Gower AJ (1990) Age-related changes in spontaneous behavior and learning in NMRI mice from maturity to middle age. Physiol Behav 47:1137–1144. 10.1016/0031-9384(90)90364-a 2395918

[B55] Li Y, Yui D, Luikart BW, McKay RM, Li Y, Rubenstein JL, Parada LF (2012) Conditional ablation of brain-derived neurotrophic factor-TrkB signaling impairs striatal neuron development. Proc Natl Acad Sci USA 109:15491–15496. 10.1073/pnas.1212899109 22949667 PMC3458400

[B56] Llano DA, Sherman SM (2008) Evidence for nonreciprocal organization of the mouse auditory thalamocortical-corticothalamic projection systems. J Comp Neurol 507:1209–1227. 10.1002/cne.21602 18181153

[B57] Lommatzsch M, Zingler D, Schuhbaeck K, Schloetcke K, Zingler C, Schuff-Werner P, Virchow JC (2005) The impact of age, weight and gender on BDNF levels in human platelets and plasma. Neurobiol Aging 26:115–123. 10.1016/j.neurobiolaging.2004.03.002 15585351

[B58] Lu H, Park H, Poo MM (2014) Spike-timing-dependent BDNF secretion and synaptic plasticity. Philos Trans R Soc Lond B Biol Sci 369:20130132. 10.1098/rstb.2013.0132 24298135 PMC3843865

[B59] Makino H, Hwang EJ, Hedrick NG, Komiyama T (2016) Circuit mechanisms of sensorimotor learning. Neuron 92:705–721. 10.1016/j.neuron.2016.10.029 27883902 PMC5131723

[B60] Maltese M, Stanic J, Tassone A, Sciamanna G, Ponterio G, Vanni V, Martella G, Imbriani P, Bonsi P, Mercuri NB, Gardoni F, Pisani A (2018) Early structural and functional plasticity alterations in a susceptibility period of DYT1 dystonia mouse striatum. Elife 7:e33331 10.7554/eLife.33331 29504938 PMC5849413

[B61] Martin SJ, Grimwood PD, Morris RG (2000) Synaptic plasticity and memory: an evaluation of the hypothesis. Annu Rev Neurosci 23:649–711. 10.1146/annurev.neuro.23.1.649 10845078

[B62] Matsumoto T, Rauskolb S, Polack M, Klose J, Kolbeck R, Korte M, Barde YA (2008) Biosynthesis and processing of endogenous BDNF: CNS neurons store and secrete BDNF, not pro-BDNF. Nat Neurosci 11:131–133. 10.1038/nn2038 18204444

[B63] McGeorge AJ, Faull RL (1989) The organization of the projection from the cerebral cortex to the striatum in the rat. Neuroscience 29:503–537. 10.1016/0306-4522(89)90128-0 2472578

[B64] McKenna WL, Betancourt J, Larkin KA, Abrams B, Guo C, Rubenstein JL, Chen B (2011) Tbr1 and Fezf2 regulate alternate corticofugal neuronal identities during neocortical development. J Neurosci 31:549–564. 10.1523/JNEUROSCI.4131-10.2011 21228164 PMC3276402

[B65] Messaoudi E, Ying SW, Kanhema T, Croll SD, Bramham CR (2002) Brain-derived neurotrophic factor triggers transcription-dependent, late phase long-term potentiation in vivo. J Neurosci 22:7453–7461. 10.1523/JNEUROSCI.22-17-07453.2002 12196567 PMC6757978

[B66] Metz GA, Whishaw IQ (2002) Cortical and subcortical lesions impair skilled walking in the ladder rung walking test: a new task to evaluate fore- and hindlimb stepping, placing, and co-ordination. J Neurosci Methods 115:169–179. 10.1016/s0165-0270(02)00012-2 11992668

[B67] Metz GA, Whishaw IQ (2009) The ladder rung walking task: a scoring system and its practical application. J Vis Exp 28:1204. 10.3791/1204 PMC2796662 19525918

[B68] Minichiello L, Korte M, Wolfer D, Kuhn R, Unsicker K, Cestari V, Rossi-Arnaud C, Lipp HP, Bonhoeffer T, Klein R (1999) Essential role for TrkB receptors in hippocampus-mediated learning. Neuron 24:401–414. 10.1016/s0896-6273(00)80853-3 10571233

[B69] Molyneaux BJ, Arlotta P, Menezes JR, Macklis JD (2007) Neuronal subtype specification in the cerebral cortex. Nat Rev Neurosci 8:427–437. 10.1038/nrn2151 17514196

[B70] Neeper SA, Gomez-Pinilla F, Choi J, Cotman C (1995) Exercise and brain neurotrophins. Nature 373:109. 10.1038/373109a0 7816089

[B71] Neeper SA, Gomez-Pinilla F, Choi J, Cotman CW (1996) Physical activity increases mRNA for brain-derived neurotrophic factor and nerve growth factor in rat brain. Brain Res 726:49–56. 8836544

[B72] Nieto M, Monuki ES, Tang H, Imitola J, Haubst N, Khoury SJ, Cunningham J, Gotz M, Walsh CA (2004) Expression of Cux-1 and Cux-2 in the subventricular zone and upper layers II-IV of the cerebral cortex. J Comp Neurol 479:168–180. 10.1002/cne.20322 15452856

[B73] Park H, Popescu A, Poo MM (2014) Essential role of presynaptic NMDA receptors in activity-dependent BDNF secretion and corticostriatal LTP. Neuron 84:1009–1022. 10.1016/j.neuron.2014.10.045 25467984 PMC4465395

[B74] Patterson SL, Abel T, Deuel TA, Martin KC, Rose JC, Kandel ER (1996) Recombinant BDNF rescues deficits in basal synaptic transmission and hippocampal LTP in BDNF knockout mice. Neuron 16:1137–1145. 10.1016/s0896-6273(00)80140-3 8663990

[B75] Perez FA, Palmiter RD (2005) Parkin-deficient mice are not a robust model of parkinsonism. Proc Natl Acad Sci USA 102:2174–2179. 10.1073/pnas.0409598102 15684050 PMC548311

[B76] Peters AJ, Chen SX, Komiyama T (2014) Emergence of reproducible spatiotemporal activity during motor learning. Nature 510:263–267. 10.1038/nature13235 24805237

[B77] Phillips HS, Hains JM, Laramee GR, Rosenthal A, Winslow JW (1990) Widespread expression of BDNF but not NT3 by target areas of basal forebrain cholinergic neurons. Science 250:290–294. 10.1126/science.1688328 1688328

[B78] Plotkin JL, Day M, Peterson JD, Xie Z, Kress GJ, Rafalovich I, Kondapalli J, Gertler TS, Flajolet M, Greengard P, Stavarache M, Kaplitt MG, Rosinski J, Chan CS, Surmeier DJ (2014) Impaired TrkB receptor signaling underlies corticostriatal dysfunction in Huntington's disease. Neuron 83:178–188. 10.1016/j.neuron.2014.05.032 24991961 PMC4131293

[B79] Rasmussen P, Brassard P, Adser H, Pedersen MV, Leick L, Hart E, Secher NH, Pedersen BK, Pilegaard H (2009) Evidence for a release of brain-derived neurotrophic factor from the brain during exercise. Exp Physiol 94:1062–1069. 10.1113/expphysiol.2009.048512 19666694

[B80] Rauskolb S, Zagrebelsky M, Dreznjak A, Deogracias R, Matsumoto T, Wiese S, Erne B, Sendtner M, Schaeren-Wiemers N, Korte M, Barde YA (2010) Global deprivation of brain-derived neurotrophic factor in the CNS reveals an area-specific requirement for dendritic growth. J Neurosci 30:1739–1749. 10.1523/JNEUROSCI.5100-09.2010 20130183 PMC6633992

[B81] Sakamoto T, Arissian K, Asanuma H (1989) Functional role of the sensory cortex in learning motor skills in cats. Brain Res 503:258–264. 10.1016/0006-8993(89)91672-7 2605518

[B82] Schuman EM (1999) Neurotrophin regulation of synaptic transmission. Curr Opin Neurobiol 9:105–109. 10.1016/s0959-4388(99)80013-0 10072368

[B83] Schweizer U, Gunnersen J, Karch C, Wiese S, Holtmann B, Takeda K, Akira S, Sendtner M (2002) Conditional gene ablation of Stat3 reveals differential signaling requirements for survival of motoneurons during development and after nerve injury in the adult. J Cell Biol 156:287–297. 10.1083/jcb.200107009 11807093 PMC2199226

[B84] Sheng MJ, Lu D, Shen ZM, Poo MM (2019) Emergence of stable striatal D1R and D2R neuronal ensembles with distinct firing sequence during motor learning. Proc Natl Acad Sci USA 116:11038–11047. 10.1073/pnas.1901712116 31072930 PMC6561210

[B85] Shiotsuki H, Yoshimi K, Shimo Y, Funayama M, Takamatsu Y, Ikeda K, Takahashi R, Kitazawa S, Hattori N (2010) A rotarod test for evaluation of motor skill learning. J Neurosci Methods 189:180–185. 10.1016/j.jneumeth.2010.03.026 20359499

[B86] Skriver K, Roig M, Lundbye-Jensen J, Pingel J, Helge JW, Kiens B, Nielsen JB (2014) Acute exercise improves motor memory: exploring potential biomarkers. Neurobiol Learn Mem 116:46–58. 10.1016/j.nlm.2014.08.004 25128877

[B87] Strand AD, Baquet ZC, Aragaki AK, Holmans P, Yang L, Cleren C, Beal MF, Jones L, Kooperberg C, Olson JM, Jones KR (2007) Expression profiling of Huntington's disease models suggests that brain-derived neurotrophic factor depletion plays a major role in striatal degeneration. J Neurosci 27:11758–11768. 10.1523/JNEUROSCI.2461-07.2007 17959817 PMC6673215

[B88] Thomson AM (2010) Neocortical layer 6, a review. Front Neuroanat 4:13. 10.3389/fnana.2010.00013 20556241 PMC2885865

[B89] Voelcker-Rehage C, Alberts JL (2007) Effect of motor practice on dual-task performance in older adults. J Gerontol B Psychol Sci Soc Sci 62:P141–P148. 10.1093/geronb/62.3.p141 17507581

[B90] Vondran MW, Clinton-Luke P, Honeywell JZ, Dreyfus CF (2010) BDNF+/- mice exhibit deficits in oligodendrocyte lineage cells of the basal forebrain. Glia 58:848–856. 10.1002/glia.20969 20091777 PMC2851835

[B91] Webster KE (1961) Cortico-striate interrelations in the albino rat. J Anat 95:532–544. 14005491 PMC1244066

[B92] West MO, Carelli RM, Pomerantz M, Cohen SM, Gardner JP, Chapin JK, Woodward DJ (1990) A region in the dorsolateral striatum of the rat exhibiting single-unit correlations with specific locomotor limb movements. J Neurophysiol 64:1233–1246. 10.1152/jn.1990.64.4.1233 2258744

[B93] Wetmore C, Ernfors P, Persson H, Olson L (1990) Localization of brain-derived neurotrophic factor mRNA to neurons in the brain by in situ hybridization. Exp Neurol 109:141–152. 10.1016/0014-4886(90)90068-4 2379553

[B94] Xiao J, Wong AW, Willingham MM, van den Buuse M, Kilpatrick TJ, Murray SS (2010) Brain-derived neurotrophic factor promotes central nervous system myelination via a direct effect upon oligodendrocytes. Neurosignals 18:186–202. 10.1159/000323170 21242670

[B95] Xu T, Yu X, Perlik AJ, Tobin WF, Zweig JA, Tennant K, Jones T, Zuo Y (2009) Rapid formation and selective stabilization of synapses for enduring motor memories. Nature 462:915–919. 10.1038/nature08389 19946267 PMC2844762

[B96] Yan Q, Rosenfeld RD, Matheson CR, Ha weekins N, Lopez OT, Bennett L, Welcher AA (1997) Expression of brain-derived neurotrophic factor protein in the adult rat central nervous system. Neuroscience 78:431–448. 10.1016/s0306-4522(96)00613-6 9145800

[B97] Yin HH, Knowlton BJ (2006) The role of the basal ganglia in habit formation. Nat Rev Neurosci 7:464–476. 10.1038/nrn1919 16715055

[B98] Ying SW, Futter M, Rosenblum K, Webber MJ, Hunt SP, Bliss TV, Bramham CR (2002) Brain-derived neurotrophic factor induces long-term potentiation in intact adult hippocampus: requirement for ERK activation coupled to CREB and upregulation of Arc synthesis. J Neurosci 22:1532–1540. 10.1523/JNEUROSCI.22-05-01532.2002 11880483 PMC6758896

[B99] Yumiya H, Ghez C (1984) Specialized subregions in the cat motor cortex: anatomical demonstration of differential projections to rostral and caudal sectors. Exp Brain Res 53:259–276. 10.1007/BF00238155 6200347

[B100] Zhang MD, Barde S, Yang T, Lei B, Eriksson LI, Mathew JP, Andreska T, Akassoglou K, Harkany T, Hokfelt TG, Terrando N (2016) Orthopedic surgery modulates neuropeptides and BDNF expression at the spinal and hippocampal levels. Proc Natl Acad Sci USA 113:E6686–E6695. 10.1073/pnas.1614017113 27791037 PMC5086993

[B101] Zunino G, Messina A, Sgado P, Baj G, Casarosa S, Bozzi Y (2016) Brain-derived neurotrophic factor signaling is altered in the forebrain of Engrailed-2 knockout mice. Neuroscience 324:252–261. 10.1016/j.neuroscience.2016.03.023 26987954

