# Supplementary figures and images for: Induction of BDNF Expression in Layer II/III and Layer V Neurons of the Motor Cortex Is Essential for Motor Learning
Source: J Neurosci. 2020 Aug 12;40(33):6289–308. doi: 10.1523/JNEUROSCI.0288-20.2020 (PMC7424868; doi:10.1523/JNEUROSCI.0288-20.2020)

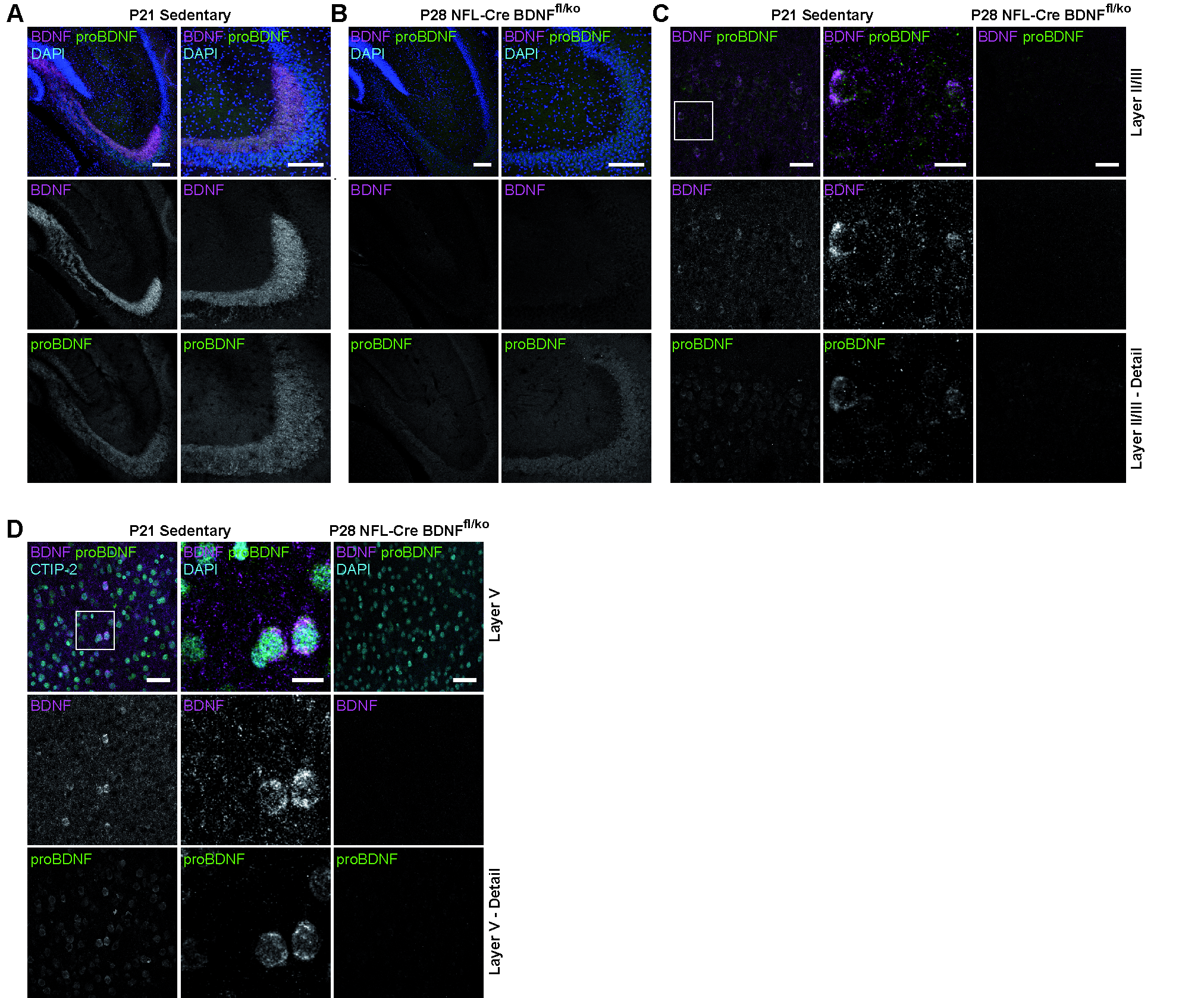

Supplement: Figure 3-1 — Detection of BDNF and pro-BDNF in mouse hippocampus and layer II/III & V motor cortex: A) BDNF and pro-BDNF-IR detected with mAb#9 antibody and a rabbit polyclonal antiserum against the BDNF pro-domain (ANT-006) in P21 WT C57Bl6/J hippocampus. Pro- and mature BDNF are expressed in mossy fiber projections and single CA3 pyramidal neurons. B) BDNF and pro-BDNF-IR in P28 NFL-Cre BDNFfl/ko derived hippocampus shows absence of mature BDNF-IR and weak pro-BDNF-IR background noise. C-D) BDNF and pro-BDNF-IR in layer II/III (C) and layer V (D) motor cortex from P21 sedentary control or P28 NFL-Cre BDNFfl/ko mice. Both isoforms are detectable in cell bodies of cortical projection neurons in WT, while immunoreactivity for both antibodies is drastically reduced in NFL-Cre BDNFfl/ko motor cortex. Raw data are provided in Table 2 - Transparent Reporting. Image type: maximum intensity projection; Scale bar: A, B) 150 µm, C, D) 50 µm overview; 15 µm detail image. Download Figure 3-1, TIF file. [file ns-JN-RM-0288-20-s03.tif]

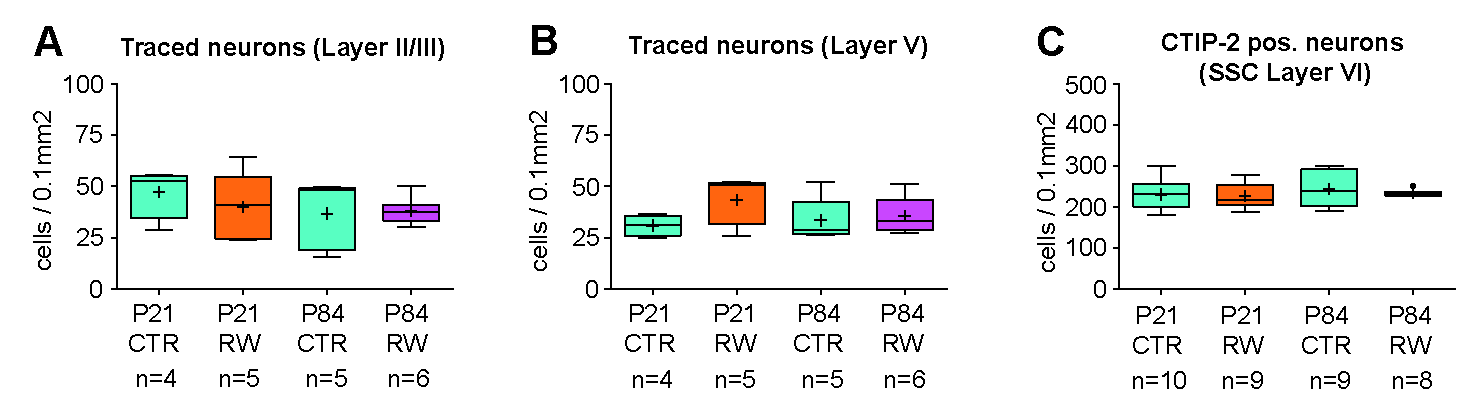

Supplement: Figure 4-2 — Variance of traced neurons in motor cortex of different groups of mice: A, B) Quantification of tracer positive neurons per area in layers II/III and V motor cortex reveals no significant difference between the four groups. C) CTIP-2 expression in layer VI of somatosensory cortex is not altered by physical activity or age. Statistical analysis: A-C) One-way ANOVA, Tukey multiple comparison post-test (A: F(3, 16)=0.5457, p = 0.6581, ANOVA; B: F(3, 16)=1.490, p = 0.2552, ANOVA; C: F(3, 32)=0.4924, p = 0.6901, ANOVA). Data are presented as box and whiskers (Tukey), “+” indicates mean, vertical line median, outliers shown as black dots; n number indicates the number of independent animals used for the analysis. Raw data are provided in Figure 4-6 - Source data and Table 2 - Transparent Reporting. Download Figure 4-2, TIF file. [file ns-JN-RM-0288-20-s05.tif]

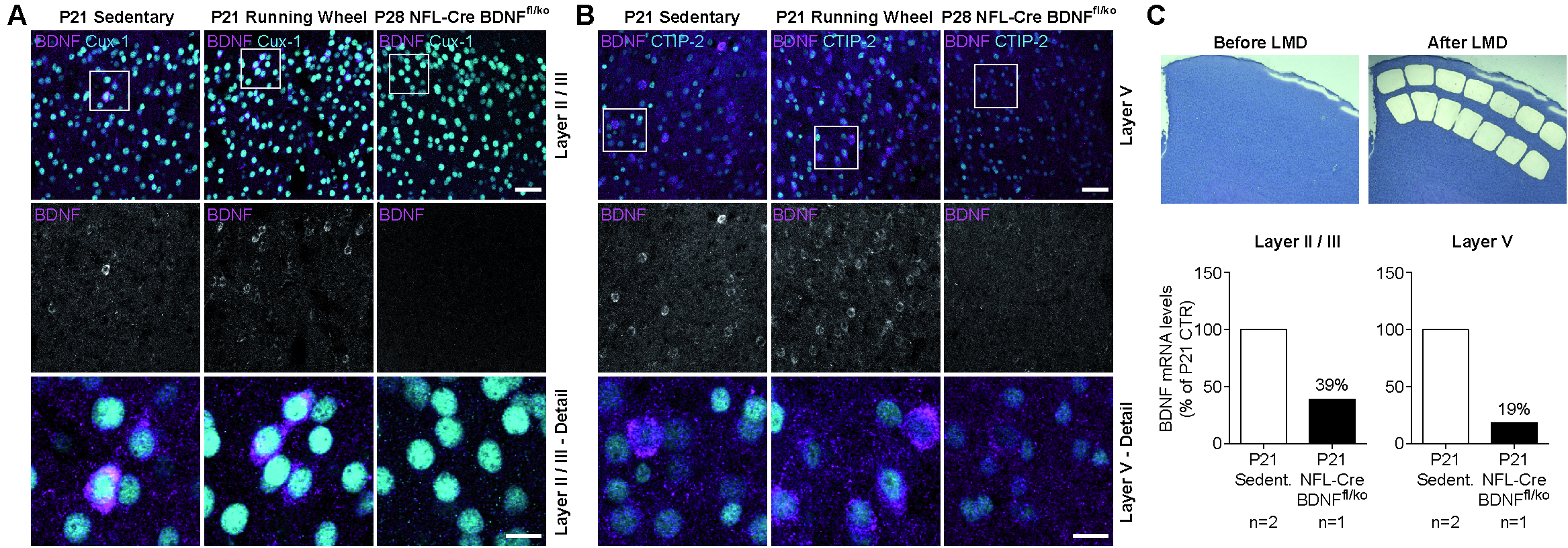

Supplement: Figure 4-3 — Cortical layer-specific alterations in BDNF protein and mRNA levels after physical exercise and conditional BDNF ablation: BDNF-IR in layers II/III (A) and layer V (B) motor cortex. P21 sedentary mice (left column), 72 h voluntary running-wheel exercise (middle column) and NFL-Cre BDNFfl/ko mice (right column). C) Images show a representative toluidine blue-stained coronal brain section of motor cortex, used for LMD of layers II/III and V for qRT-PCR analysis. qRT-PCR for BDNF, normalized to GAPDH revealed a reduction of BDNF mRNA by ∼60% in layer II-III and ∼80% in layer V motor cortex in NFL-Cre BDNFfl/ko mice. Data are presented as levels relative to wild-type controls (CTR) in bar graphs for representative visualization of BDNF mRNA reduction (no statistical test was used, because of low n-number); n number indicated below. Raw data are provided in Figure 4-7 - Source data and Table 2 - Transparent Reporting. Image type: A, B) maximum intensity projection; Scale bar: A, B) 50 µm; 15 µm (detail). Download Figure 4-3, TIF file. [file ns-JN-RM-0288-20-s06.tif]

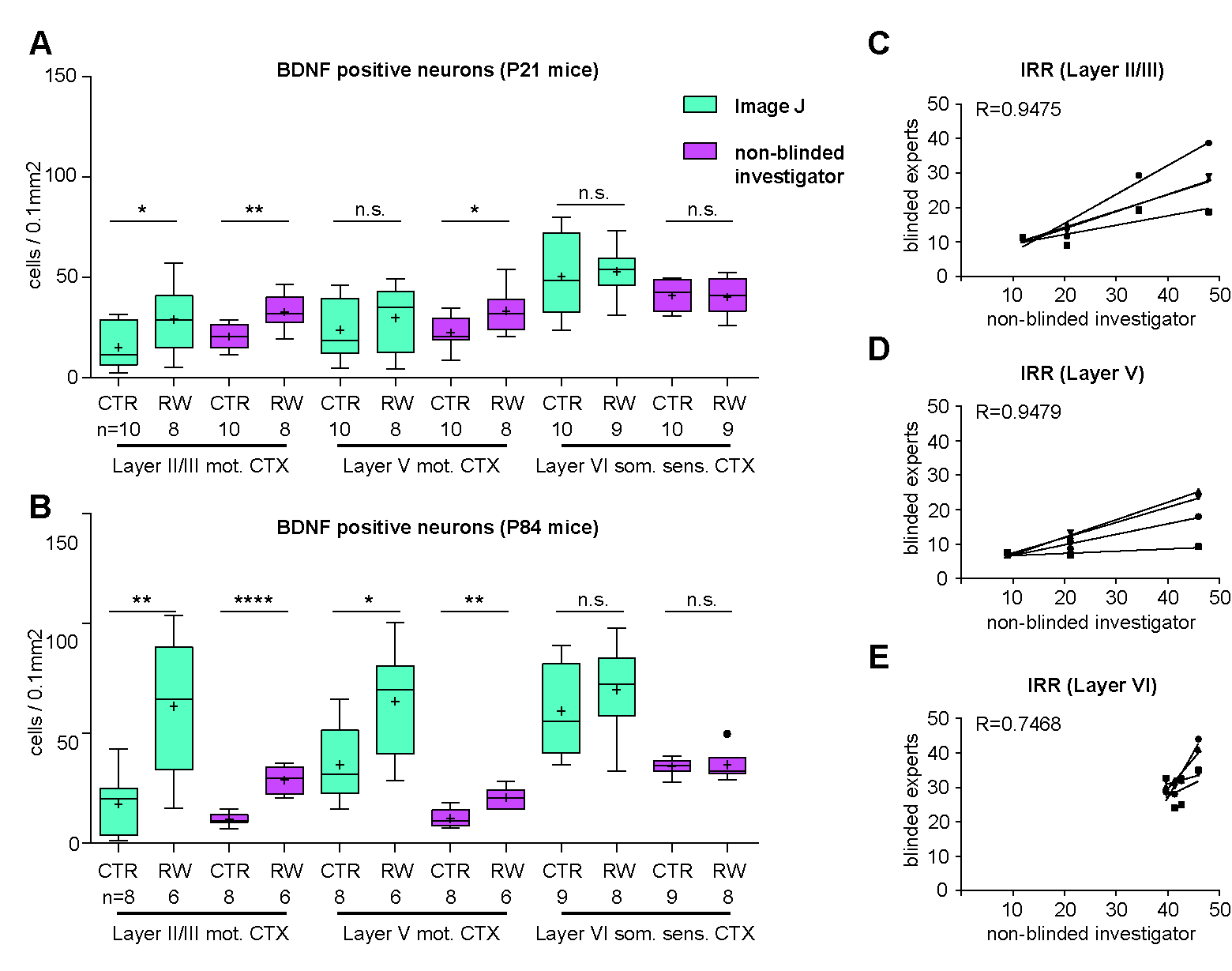

Supplement: Figure 4-4 — Quantification of BDNF expression in cortical neurons by different experimenters and automatic quantification using ImageJ: A, B) Quantification of BDNF-expressing neurons in P21 (A) or P84 (B) cortex using ImageJ versus manual counting by non-blinded investigator (n number indicated below). C-E) Correlation analysis between the non-blinded investigator and 4 blinded experts for BDNF-positive cell counts in layers II/III (C), V (D) motor cortex and layer VI somatosensory cortex (E). 3 random sample images were analyzed for each of the following conditions: P21 CTR, P21 RW, P84 CTR, P84 RW. Statistical analysis: A, B) unpaired t test between corresponding pairs (A: LII/III: t = 2.147, p = 0.0475, investigator: t = 3.630, p = 0.0023, LV: t = 0.8230, p = 0.4226, investigator: t = 2.487, p = 0.0243; LVI: t = 0.3224, p = 0.7511, investigator: t = 0.2480, p = 0.8071; B: LII/III: t = 3.598, p = 0.0037, investigator: t = 7.212, p < 0.0001, LV: t = 2.559, p = 0.0251, investigator: t = 3.892, p = 0.0021; LVI: t = 0.9821, p = 0.3416), P84 somatosensory CTX layer VI of non-blinded investigator - Mann–Whitney test (Mann–Whitney U 34.00, p = 0.8619). C-E) Linear regression and correlation analysis, Pearson's R value for correlation is indicated in each graph. Data are presented as box and whiskers (Tukey), “+” indicates mean, vertical line median, outliers shown as black dots; n number indicates the number of independent animals used for the analysis. Raw data are provided in Figure 4-8 - Source data and Table 2 - Transparent Reporting. Download Figure 4-4, TIF file. [file ns-JN-RM-0288-20-s07.tif]

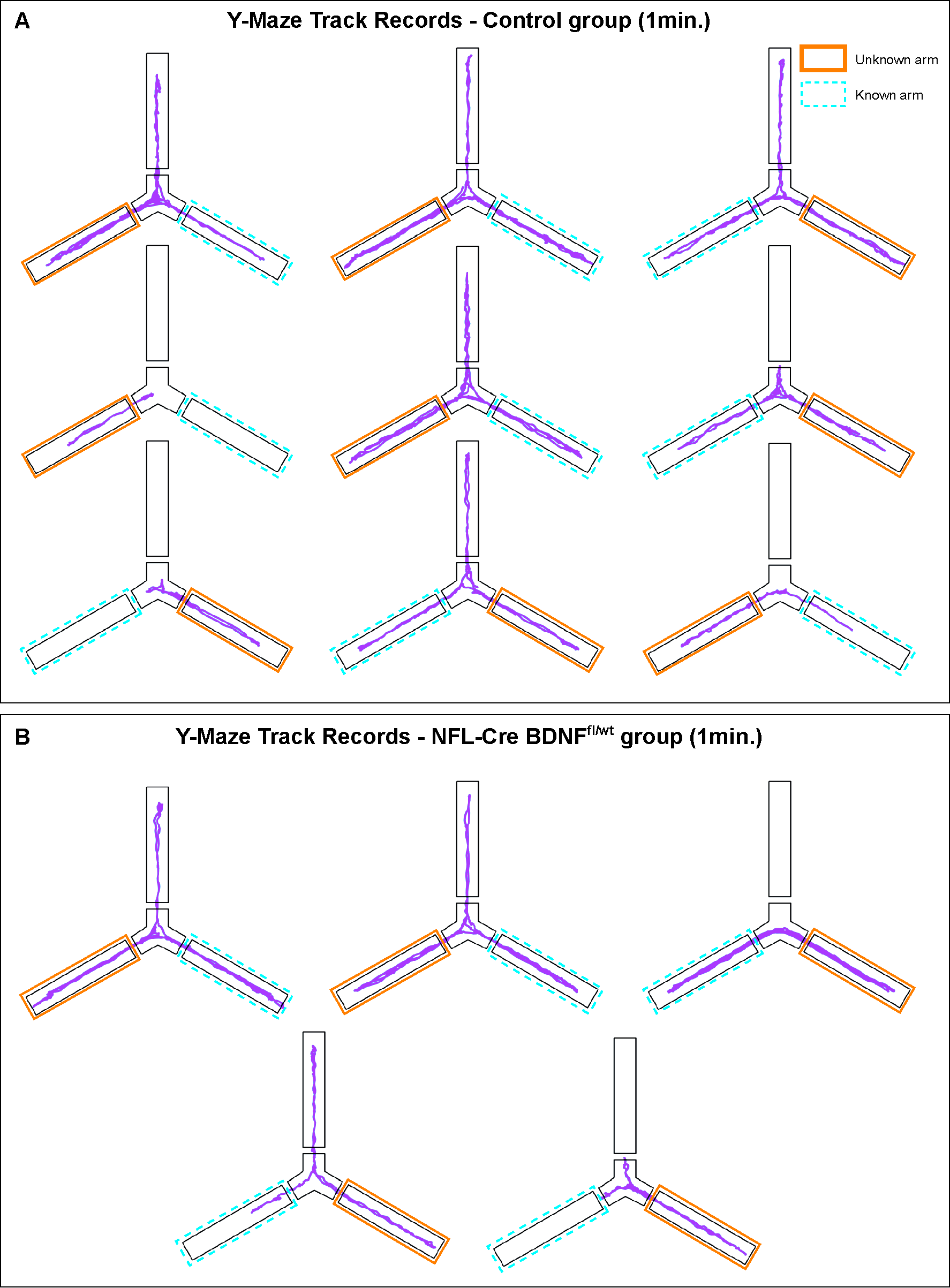

Supplement: Figure 8-1 — Y-Maze track records during the first minute after opening of the closed arm in the spatial reference memory test run: Documentation of tracks for control mice (A) and NFL-Cre BDNFfl/wt mice (B). Animals were placed in the start-arm (top) and were able to choose between the known arm (blue) and the unknown arm (orange). Download Figure 8-1, TIF file. [file ns-JN-RM-0288-20-s16.tif]
